# Cosmological reconstruction and energy constraints in generalized Gauss–Bonnet-scalar–kinetic–matter couplings

**DOI:** 10.1038/s41598-020-75067-9

**Published:** 2020-10-22

**Authors:** Adam Z. Kaczmarek, Dominik Szczęśniak

**Affiliations:** 1 Institute of Physics, Częstochowa University of Technology, 19 Armii Krajowej Ave., 42200 Czestochowa, Poland; 2 Department of Chemistry, Purdue University, 560 Oval Dr., West Lafayette, IN 47907 USA; 3 Department of Theoretical Physics, Faculty of Science and Technology, Jan Długosz University in Częstochowa, 13/15 Armii Krajowej Ave., 42200 Czestochowa, Poland

**Keywords:** Cosmology, General relativity and gravity

## Abstract

Recently introduced $f(\mathcal {G},T)$ theory is generalized by adding dependence on the arbitrary scalar field $\phi$ and its kinetic term $(\nabla \phi )^2$, to explore non-minimal interactions between geometry, scalar and matter fields in context of the Gauss–Bonnet theories. The field equations for the resulting $f\left( \mathcal {G},\phi ,(\nabla \phi )^2,T\right)$ theory are obtained and show that particles follow non-geodesic trajectories in a perfect fluid surrounding. The energy conditions in the Friedmann–Lemaître–Robertson–Walker (FLRW) spacetime are discussed for the generic function $f\left( \mathcal {G},\phi ,(\nabla \phi )^2,T\right)$. As an application of the introduced extensions, using the reconstruction techniques we obtain functions that satisfy common cosmological models, along with the equations describing energy conditions for the reconstructed $f\left( \mathcal {G},\phi ,(\nabla \phi )^2,T\right)$ gravity. The detailed discussion of the energy conditions for the de Sitter and power-law spacetimes is provided in terms of the fixed kinetic term i.e. in the $f\left( \mathcal {G},\phi ,T\right)$ case. Moreover, in order to check viability of the reconstructed models, we discuss the energy conditions in the specific cases, namely the $f(R,\phi ,(\nabla \phi )^2)$ and $f=\gamma (\phi ,X)\mathcal {G}+\mu T^{1/2}$ approaches. We show, that for the appropriate choice of parameters and constants, the energy conditions can be satisfied for the discussed scenarios.

## Introduction

The accelerated expansion of our Universe is one of the biggest challenges in theoretical physics. In particular, recent experimental data turns attention of the scientific community to the dark energy (DE) as a new form of matter, that drives accelerated phase in cosmology^1,2^. However, the most popular $\Lambda$-cold dark matter ($\Lambda$-CDM) model suffers from cosmological constant problem and from various observational issues e.g. the missing satellites and the cusp problem^3^. As a result, a new ways to resolve the DE mystery are desirable. Over the years, a large number of possible models and candidates for the dark energy were introduced and studied extensively^3,4^. One of the possible explanations of the DE problem originates from the potential breakdown of the Einstein’s general relativity (GR) at the large cosmological scales^4,5^. This leads to so called modified theories of gravity, where alternative gravitational actions are introduced^3,6^. Such approach achieved significant attention in studying cosmic expansion and other issues in cosmology^7^. Moreover, important attempts were made to tackle unification of current expansionary era with inflationary epoch^8^. For essential reviews, please see^3,4,9,10^. In these models, source of the dark energy comes from the modified Einstein–Hilbert action, in theory avoiding need of the strange types of matter. There is a vast literature exploring different generalisations of the GR, that are achieved usually by introducing scalar invariants and their generic functions in action. One of the simplest and most popular example is the *f*(*R*) gravity, where *R* is the Ricci scalar introduced by Buchdachl^11^. Its viability in cosmological context, as well as stability, were widely studied^12,13^. It was shown, that dark energy may be in fact geometric effect coming from special choice of the *f*(*R*) function^14,15^. Another interesting theory is the $f(\mathcal {G})$ gravity, where $\mathcal {G}$ represents the Gauss–Bonnet invariant^16^. It is worth to mention that $\mathcal {G}$ is the topological invariant in four dimensions and is important in higher dimensional brane-world descriptions of gravity. This theory is consistent with solar system tests and may describe accelerated expansion of the Universe. Both of these models were recently extended to the $f(R,\mathcal {G})$ theory and studied in context of the DE^17,18^.

Another class of proposals is based on the coupling between matter and geometry, for example the *f*(*R*, *T*), $f(\mathcal {G},T)$ and $f(R,\mathcal {L}_m)$ theories, where *T* is trace of the energy momentum tensor and $\mathcal {L}_m$ is the matter Lagrangian^19–21^. These theories are characterised by non-conservation of the energy–momentum tensor. It was also shown, that in presence of the geometry–matter coupling, test particles will experience extra force, orthogonal to their four velocities, that leads to the non-geodesic motion^22,23^. Interestingly, Nojiri et al.^24^ discussed nonminimal coupling of the $\mathcal {L}_m$ to the *f*(*R*) and $f(\mathcal {G})$ theories, showing that they can unify current accelerated expansion with the era of inflation. Moreover, thermodynamical interpretation was recently introduced; additional terms from curvature–matter coupling may be a way to describe particle production where gravitational field is the source of particles^25^. While searching for alternative matter–geometry couplings, Haghani et al.^26^ and Odintsov et al.^27^ introduced the $f(R,T,R_{\mu \nu }T^{\mu \nu })$ theory, where besides trace *T*, matter couples with geometry by contraction of the Ricci and energy–momentum tensors ($R_{\mu \nu }T^{\mu \nu }$). Another approach was recently introduced by Xu and collaborators^28^; the *f*(*Q*, *T*) theory, where trace of the energy–momentum tensor is coupled with the non-metricity *Q*. For further and more complex discussion on the geometry–matter couplings we refer reader to^6,29^.

Slightly different idea was first considered by Jordan et al.^30,31^. In the so called scalar–tensor theories of gravity, scalar field $\phi$ is included in gravitational action as a realisation of the Mach’s principle in general relativity^32^. The scalar fields seems to be crucial in explaining inflationary phase of cosmic scenario. In modern literature, the scalar-tensor idea is often combined with the approach based on scalar invariants^3,9^. Besides the $f(R,\phi )$ theory, the scalar-Gauss–Bonnet gravity (or general $f(\mathcal {G},\phi )$ gravity) is very interesting and promising, due to the relation with low-energy effective action of the string theory^33,34^. The dynamics of viable inflationary models in the scalar-Gauss–Bonnet theory were recently studied by Odintsov and Oikonomou^35^. Another curious proposal was recently presented by Bahamonde et al.^36^. The generic $f(R,\phi )$ function was generalised to the $f(R,\phi ,X)$ theory, where $X=(\nabla \phi )^2$ is the kinetic term. By using the reconstruction techniques, Authors reproduced late-time acceleration and the $\Lambda$CDM model for simple actions. Spherical symmetric solutions and linear stability of the thick branes were studied in context of the $f(R,\phi ,X)$ gravity^37,38^; as well as inflation^39^. Another interesting theory, the $f(R,R_{\mu \nu }R^{\mu \nu },\phi )$ approach, has been introduced in context of the gravitational waves propagation by Lambiase et al.^40^.

Furthermore, possible geometry–matter–scalar fields couplings were also investigated. They can appear in low energy effective limits of string theories^41,42^ or compactified Kaluza–Klein models^43^ and may be useful in discussing interactions between dark energy and dark matter^44^. Other approaches to matter–scalar field coupling were also investigated^45–47^. Moreover, the so-called *chameleon* mechanism is worth emphasis. In this approach, the scalar field blends in high density regions with its environment, being in fact invisible^48^. This is consequence of the *chameleon* scalar field coupling with matter^48^. This led to introduction of the $f(R,\phi ,(\nabla \phi )^2,\mathcal {L}_m)$ gravity^49^. In addition, axion field coupling to matter and geometry may also be interesting^50–52^. Furthermore, Authors of^53^ suggested that shortcomings and issues of the standard *f*(*R*, *T*) gravity may be fixed by inclusion of scalar fields in considerations.

The energy conditions play crucial role in the general relativity^54,55^. Einstein’s field equations admit vast number of possible solutions—since geometric side of solution is proper, behaviour of the matter content is a possible way to distinguish physical solutions. To do so, specific constraints are imposed on matter distribution and energy momentum tensor. They originate from the Raychaudhuri equations and are invocations of the energy positiveness and the gravity attractiveness. Energy conditions are extremely important in the black hole physics, as they are laying foundations of the singularity theorems and play crucial role in the black hole’s thermodynamics^55,56^. There are four fundamental energy conditions^54^:

- the null energy condition (NEC)—asserts that matter density is positive along null (lightlike) curves,
- the weak energy condition (WEC)—states that along timelike curves energy density stays positive,
- the dominant energy condition (DEC)—states that the speed of energy–mass flow cannot exceed speed of light,
- the strong energy condition (SEC)—assures that matter gravitates towards matter i.e. gravity is focusing force.

The energy conditions has been studied previously in extended theories of gravity, like the scalar-tensor, *f*(*R*) and $f(\mathcal {G})$ theories^57–64^. In context of the FLRW cosmology they were discussed also in the generalised teleparallel $f(\mathcal {T})$ gravity, where $\mathcal {T}$ is the torsion scalar^65^. For geometry–matter coupling, the energy conditions have been considered in context of the *f*(*R*, *T*), $f(\mathcal {G,T})$ and $f(R,T,R_{\mu \nu }T^{\mu \nu })$ theories^20,66,67^. Zubair and Kousar^68^ investigated these conditions for the $f(R,R_{\mu \nu }R^{\mu \nu },\phi )$ theory and for specific well-known $f(R,\phi )$ models; showing that for NEC validity, certain conditions on parameters in the $f(R,\phi )$ case should be imposed.

In this paper, we introduce formalism of the $f\left( \mathcal {G},\phi ,(\nabla \phi )^2,T\right)$ gravity, where $\mathcal {G}$, $\phi$, $(\nabla \phi )^2$, *T* are Gauss–Bonnet term, scalar field, kinetic term and trace of energy–momentum tensor, respectively. We intent to extend previously considered ideas of scalar field and matter couplings in the *f*(*R*) framework to the GB case. As we indicated before, there is vast literature exploring scalar fields in the GB theories and recently introduced *f*(*G*, *T*) theory grows on popularity as well^20,69–74^. We derive field equations for this theory and show that test particles follow non-geodesic paths. In case of the FLRW metric, we overview energy conditions for the generic function *f*, and discuss them for specific models using reconstruction techniques. Additionally, we discuss energy conditions of the $f(R,\phi ,(\nabla \phi )^2)$ gravity for models presented by^36^. We introduce $\gamma (\phi ,(\nabla \phi )^2)\mathcal {G}+g(T)$ function in the power law scenario and study its energy conditions, showing that NEC nad WEC can be satisfied for suitable choice of parameters and integration constants.

In present work, $\nabla _\alpha$ denotes usual covariant derivative with respect to $\alpha$-th component ($x^\alpha =(x^0,x^1,x^2,x^3)=(x^0,x^i)$), while $\partial _\alpha =\frac{\partial }{\partial x^\alpha }$ is usual partial derivative. We use units with $c=1$ and signature of metric $(+---)$. The gravitational coupling is denoted as $\chi$. This paper is organized as follows. In the next section we introduce field equation of the $f\left( \mathcal {G},\phi ,(\nabla \phi )^2,T\right)$ theory and discuss energy conservation with application to the cosmological perfect fluid. Section 3 is devoted to the energy conditions for the cosmological perfect fluid in the $f\left( \mathcal {G},\phi ,(\nabla \phi )^2,T\right)$ theory. In, Sect. 4 we use reconstruction technique and analyze energy conditions in the obtained models, while in Sect. 5 we discuss energy conditions of the $f(R,\phi ,X)$ gravity theory and for the $\gamma (\phi ,X)\mathcal {G}+g(T)$ model. Last section is left for conclusions and summary.

## Theoretical model

In order to formulate equations of the theory, we start from the action in the following form:

1 $$\begin{aligned} S=\frac{1}{2\chi }\int d^4x\sqrt{-g}\left[ R+f\left( \mathcal {G},\phi ,(\nabla \phi )^2,T\right) \right] + \int d^4x\sqrt{-g} \mathcal {L}_{m}(g,\psi ), \end{aligned}$$

where $R=R^\mu _\mu$ denotes Ricci scalar and $f\left( \mathcal {G},\phi ,(\nabla \phi )^2,T\right)$ is analytical (Taylor expanding) function of the Gauss–Bonnet term $\mathcal {G}=R^2-4R^{\mu \nu }_{\quad \mu \nu }+R^{\quad \quad \mu \nu \alpha \beta }_{\mu \nu \alpha \beta }$. The scalar field $\phi$, its kinetic term $(\nabla \phi )^2=g^{\mu \nu }\nabla _\mu \phi \nabla _\nu \phi$, and trace of the energy–momentum tensor $T=T^\mu _\mu$; $\mathcal {L}_{m}$ is the matter Lagrangian density which depends on metric tensor $g_{\mu \nu }$ and matter fields $\psi$. The energy–momentum tensor is defined as:

2 $$\begin{aligned} T_{\mu \nu }=\frac{-2}{\sqrt{-g}}\frac{\delta (\sqrt{-g}\mathcal {L}_m)}{\delta g^{\mu \nu }}. \end{aligned}$$

Furthermore, assuming that matter distribution is independent on derivatives of the metric tensor $g_{\mu \nu }$, we obtain:

3 $$\begin{aligned} T_{\mu \nu }=g_{\mu \nu }\mathcal {L}_m-2\frac{\partial \mathcal {L}_m}{\partial g^{\mu \nu }}. \end{aligned}$$

Next, we vary gravitational action (1) with respect to the components of the metric tensor $g^{\mu \nu }$:

4 $$\begin{aligned} 0=\delta S=\frac{1}{2\chi }\int d^4x\left[ \sqrt{-g} \delta (R+f\left( \mathcal {G},\phi ,(\nabla \phi )^2,T\right) )+ (R+f\left( \mathcal {G},\phi ,(\nabla \phi )^2,T)\right) \delta \sqrt{-g} \right] +\int d^4x\sqrt{-g}\delta \left( \mathcal {L}_m(g,\psi )\right) . \end{aligned}$$

First term on the right hand side of Eq. (4) can be expanded as:

5 $$\begin{aligned}&\frac{1}{2\chi }\int d^4x\left[ \sqrt{-g} \delta \left( R+f(\mathcal {G},\phi ,(\nabla \phi )^2,T)\right) + \left( R+f(\mathcal {G},\phi ,(\nabla \phi )^2,T)\right) \delta \sqrt{-g}\right] \nonumber \\&\quad =\frac{1}{2\chi }\int d^4x\left[ \sqrt{-g} \left( \delta R+f_\mathcal {G}\delta \mathcal {G}\right) +f_{(\nabla \phi )^2}\delta (\nabla \phi )^2+f_T\delta T)+ (R+f\left( \mathcal {G},\phi ,(\nabla \phi )^2,T\right) )\delta \sqrt{-g}\right] , \end{aligned}$$

where we assumed that the scalar field is independent of the metric tensor $\delta \phi / \delta g^{\mu \nu }=0$ and thus $f_\phi \delta \phi =0$. In the above equation, function $f\left( \mathcal {G},\phi ,(\nabla \phi )^2,T\right)$ for notational brevity is denoted as *f* and $f_\mathcal {G}=\partial f/\partial \mathcal {G}$, $f_{(\nabla \phi )^2}=\partial f/ \partial (\nabla \phi )^2$, $f_T=\partial f/\partial T$ stand for the partial derivatives with respect to the arguments. Variations of particular terms are given as:

6 $$\begin{aligned} \delta \sqrt{-g}&=-\frac{1}{2}\sqrt{-g}g_{\mu \nu }\delta g^{\mu \nu },\nonumber \\ \delta T&=T_{\mu \nu }+g^{\alpha \beta }\frac{\delta T_{\alpha \beta }}{\delta g_{\mu \nu }}=T_{\mu \nu }+\Theta _{\mu \nu },\nonumber \\ \delta (\nabla \phi )^2&=\delta g^{\mu \nu }\nabla _{\mu }\phi \nabla _{\nu }\phi ,\nonumber \\ \delta \mathcal {G}&=2R\delta R-4\delta R^{\mu \nu }_{\quad \mu \nu }+\delta K,\nonumber \\ \delta R&=(R_{\mu \nu }+g_{\mu \nu }\Box -\nabla _\mu \nabla _\nu )g^{\mu \nu },\nonumber \\ \delta R^\alpha _{\mu \nu \beta }&=\nabla _\nu (\delta \Gamma ^\alpha _{\beta \mu })-\nabla _\beta (\delta \Gamma ^\alpha _{\nu \mu }) =(g_{\mu \lambda }\nabla _{[\beta }\nabla _{\nu ]}+g_{\lambda [\nu }\nabla _{\beta ]}\nabla _{\mu })\delta g^{\alpha \lambda },\nonumber \\ \delta R_{\mu \nu }&=\delta R^\alpha _{\mu \alpha \nu }, \end{aligned}$$

where $K=R^{\quad \quad \mu \nu \alpha \beta }_{ \mu \nu \alpha \beta }$ denotes Kretschmann scalar and tensor $\Theta _{\mu \nu }$ is defined as:

7 $$\begin{aligned} \Theta _{\mu \nu }=g^{\alpha \beta }\frac{\delta T_{\alpha \beta }}{\delta g_{\mu \nu }}. \end{aligned}$$

Using relations (6) together with Eq. (5), we obtain field equations for the $f\left( \mathcal {G},\phi ,(\nabla \phi )^2,T\right)$ gravity:

8 $$\begin{aligned}&G_{\mu \nu }+(T_{\mu \nu }+\Theta _{\mu \nu })f_T+\nabla _{\mu }\phi \nabla _{\nu }\phi f_{(\nabla \phi )^2}-\frac{1}{2}g_{\mu \nu }f +[2RR_{\mu \nu }-4R^\lambda _\mu R_{\lambda \nu }-4R_{\mu \alpha \nu \beta }R^{\alpha \beta }+2R^{\lambda \ \alpha \beta }_{\ \mu }R_{\nu \lambda \beta \alpha }]f_\mathcal {G}\nonumber \\&\quad +\left[ 2Rg_{\mu \nu }\Box -2R\nabla _\mu \nabla _\nu -4g_{\mu \nu }R^{\alpha \beta }\nabla _\alpha \nabla _\beta -4R_{\mu \nu }\Box +4R^\lambda _\mu \nabla _\nu \nabla _\lambda +4R^\lambda _\nu \nabla _\mu \nabla _\lambda +4R_{\mu \alpha \nu \beta }\nabla ^\alpha \nabla ^\beta \right] f_\mathcal {G} =\chi T_{\mu \nu }. \end{aligned}$$

Trace of Eq. (8) is given by:

9 $$\begin{aligned} R-(\nabla \phi )^2f_{(\nabla \phi )^2}-(T+\Theta )f_T+2f+2\mathcal {G}f_\mathcal {G}-2R\Box f_\mathcal {G} +4R^{\mu \nu }\nabla _\mu \nabla _\nu f_\mathcal {G}+\chi T=0, \end{aligned}$$

with $\Theta =\Theta ^\mu _\mu$. The gravitational field equations can be rewritten in a form identical to the Einstein’s field equations:

10 $$\begin{aligned} G_{\mu \nu }=\chi T_{\mu \nu }^{eff}=\chi (T_{\mu \nu }+T_{\mu \nu }^{grav}), \end{aligned}$$

where $T_{\mu \nu }^{grav}$ is contribution coming from the $f(\mathcal {G},\phi ,(\nabla \phi )^2,T)$ term, given by:

11 $$\begin{aligned} T_{\mu \nu }^{grav}&=\frac{1}{\chi }\left[ -(T_{\mu \nu }+\Theta _{\mu \nu })f_T -\nabla _{\mu }\phi \nabla _{\nu }\phi f_{(\nabla \phi )^2}+\frac{1}{2}g_{\mu \nu }f -[2RR_{\mu \nu }-4R^\lambda _\mu R_{\lambda \nu }-4R_{\mu \alpha \nu \beta }R^{\alpha \beta }+2R^{\lambda \ \alpha \beta }_{\ \mu }R_{\nu \lambda \beta \alpha }]f_\mathcal {G}\right. \nonumber \\&\quad \left. -\left[ 2Rg_{\mu \nu }\Box -2R\nabla _\mu \nabla _\nu -4g_{\mu \nu }R^{\alpha \beta }\nabla _\alpha \nabla _\beta -4R_{\mu \nu }\Box +4R^\lambda _\mu \nabla _\nu \nabla _\lambda +4R^\lambda _\nu \nabla _\mu \nabla _\lambda +4R_{\mu \alpha \nu \beta }\nabla ^\alpha \nabla ^\beta \right] f_\mathcal {G} \right] . \end{aligned}$$

To improve context, we note that the effective energy momentum tensor is composed from the standard matter content ($T_{\mu \nu }$) and contribution coming from the considered extension of the GR ($T^{grav}_{\mu \nu }$). This tensor contains curvature terms that modifies GR, in terms of geometry, matter and scalar field corrections with couplings between them. It can be also regarded as the energy–momentum tensor of matter with the dark energy corrections^63,75^. In the perfect fluid description, besides standard matter fluid, the curvature fluid will be present. It is important to remark, that such curvature fluid may admit features not present in the standard matter. Moreover, using proper conformal transformation, one could define effective ***stress-energy tensor as $\bar{T}_{\mu \nu }^{eff}\sim \bar{T}_{\mu \nu }+\bar{T}^\varphi _{\mu \nu }$, where $\bar{T}^\varphi _{\mu \nu }$ will be contribution coming from the coupling with new scalar field (or fields) $\varphi$. It is important to remark, that the geometrical implications and the energy conditions of the effective energy–momentum tensor may vary between conformal related frames^63,75,76^.

Since, we are dealing with the scalar field, varying action (1) with respect to $\phi$ leads to the scalar equation:

12 $$\begin{aligned} \frac{1}{2}f_\phi =\Box _{(\nabla \phi )^2}\phi =\nabla _\mu f_{(\nabla \phi )^2}\nabla ^\mu \phi +f_{(\nabla \phi )^2}\Box \phi . \end{aligned}$$

This equation describes dynamics of the scalar field $\phi$ in the $f\left( \mathcal {G},\phi ,(\nabla \phi )^2,T\right)$ formalism. It is worth to note that the field equations have form similar to the $f(\mathcal {G},T)$ theory, and significant differences comes from the equation for the scalar field $\phi$. The covariant divergence of Eq. (8) gives:

13 $$\begin{aligned} \nabla ^\mu T_{\mu \nu }&=\frac{f_T}{\chi -f_T}\left[ \left( T_{\mu \nu }+\Theta _{\mu \nu }\right) \nabla ^\mu ln(f_T)-\frac{1}{2}\nabla _\nu T +\nabla ^\mu \Theta _{\mu \nu } \right] , \end{aligned}$$

and is non-zero. Now we calculate tensor $\Theta _{\mu \nu }$. From Eq. (3) we obtain:

14 $$\begin{aligned} \frac{\delta T_{\mu \nu }}{\delta g^{\alpha \beta }}=\frac{\delta g_{\mu \nu }}{\delta g^{\alpha \beta }}+ g_{\mu \nu }\frac{\partial \mathcal {L}_m}{\partial g^{\alpha \beta }}-2\frac{\partial ^2 \mathcal {L}_m}{\partial g^{\alpha \beta }\partial g^{\mu \nu }} . \end{aligned}$$

Using generalised Kronecker delta $\delta ^{\lambda \eta }_{\alpha \beta }=\delta g^{\lambda \eta }/\delta g^{\alpha \beta }$, with the relation:

15 $$\begin{aligned} \frac{\delta g_{\mu \nu }}{\delta g^{\alpha \beta }}=-g_{\mu \lambda }g_{\nu \eta }\delta ^{\lambda \eta }_{\alpha \beta }, \end{aligned}$$

and substituting Eq. (14) into Eq. (7), we obtain:

16 $$\begin{aligned} \Theta _{\mu \nu }=-2T_{\mu \nu }+g_{\mu \nu }\mathcal {L}_m-2g^{\alpha \beta }\frac{\partial ^2 \mathcal {L}_m}{\partial g^{\mu \nu }\partial g^{\alpha \beta }}. \end{aligned}$$

As additional remark we briefly discuss theory with action (1) replaced by:

17 $$\begin{aligned} S=\frac{1}{2\chi }\int d^4x\sqrt{-g}\left[ F\left( R,\mathcal {G},\phi ,(\nabla \phi )^2,T\right) \right] + \int d^4x\sqrt{-g} \mathcal {L}_{m}(g,\psi ). \end{aligned}$$

Function $F\left( R,\mathcal {G},\phi ,(\nabla \phi )^2,T\right)$ is generalised by including arbitrary dependence on the Ricci scalar *R*. Clearly, for the $f\left( \mathcal {G},\phi ,(\nabla \phi )^2,T\right) =R+F\left( R,\mathcal {G},\phi ,(\nabla \phi )^2,T\right)$ discussed theory is recovered. This action contains rich variety of known dark energy and extended gravity models such as Galileons and theories based on generic function of the Ricci scalar (e.g. *f*(*R*), $f(R,\phi ,(\nabla \phi )^2)$, $f(R,\phi )$, *f*(*R*, *T*))^12,19,36^. From action (17) one can derive field equations:

18 $$\begin{aligned}&G_{\mu \nu } F_R-\frac{1}{2}g_{\mu \nu }(F-RF_R)+(g_{\mu \nu }\Box -\nabla _\mu \nabla _\nu )F_R+[2RR_{\mu \nu }-4R^\lambda _\mu R_{\lambda \nu }-4R_{\mu \alpha \nu \beta }R^{\alpha \beta }+2R^{\lambda \ \alpha \beta }_{\ \mu }R_{\nu \lambda \beta \alpha }]F_\mathcal {G} \nonumber \\&\quad +[2Rg_{\mu \nu }\Box -2R\nabla _\mu \nabla _\nu -4g_{\mu \nu }R^{\alpha \beta }\nabla _\alpha \nabla _\beta -4R_{\mu \nu }\Box +4R^\lambda _\mu \nabla _\nu \nabla _\lambda +4R^\lambda _\nu \nabla _\mu \nabla _\lambda +4R_{\mu \alpha \nu \beta }\nabla ^\alpha \nabla ^\beta ]F_\mathcal {G}\nonumber \\&\quad +(T_{\mu \nu }+\Theta _{\mu \nu })F_T +\nabla _{\mu }\phi \nabla _{\nu }\phi F_{(\nabla \phi )^2}=\chi T_{\mu \nu }. \end{aligned}$$

Next, we discuss properties of the perfect fluid fields, that constitute matter content of the universe. The energy–momentum tensor of perfect fluid, described by the energy density $\rho$ and pressure *P*, is given by:

19 $$\begin{aligned} T_{\mu \nu }=(\rho +P)U_\mu U_\nu -Pg_{\mu \nu }, \end{aligned}$$

where $U^\mu$ is four velocity of the fluid, and satifies relation $U^\mu U_\mu =1$. The corresponding Lagrangian is $\mathcal {L}_m=-P$. Then, from Eq. (13), the expression for the $\Theta _{\mu \nu }$ tensor can be provided:

20 $$\begin{aligned} \Theta _{\mu \nu }=-2(T_{\mu \nu }+Pg_{\mu \nu }). \end{aligned}$$

Now, we discuss geodesic motion of the massive test particles moving through the perfect fluid in the discussed model. From Eq. (13), together with Eqs. (19) and (20), the conservation equation for the energy–momentum tensor is:

21 $$\begin{aligned}&\nabla _{\nu }(\rho +P)U^{\mu }U^{\nu }+(\rho +P)[U^{\nu }\nabla _{\nu }U^{\mu }+U^{\mu }\nabla _{\nu }U^{\nu }-\nabla ^{\mu }P]\nonumber \\&\quad =\frac{-2}{2\chi +3f_T}[T^{\mu \nu }\nabla _{\nu }f_T+(\nabla ^{\mu }P) f_T+P (\nabla ^{\mu }f_T)]. \end{aligned}$$

Multiplying above equation by the projection operator $h_{\eta \mu }=\delta _{\eta \mu }-U_\eta U_\mu$, where $U^\mu h_{\eta \mu }=0$ and $U^{\mu }\nabla _{\nu }U_{\mu }=0$, gives:

22 $$\begin{aligned} g_{\mu \eta }U^{\nu }\nabla _{\nu }U^{\mu }=\frac{(2\chi +f_T)}{(\rho +P)(2\chi +3f_T)}\nabla _{\nu }h^{\nu }_{\eta }, \end{aligned}$$

where we have used the fact that $h_{\mu \eta }T^{\mu \nu }=-Ph^{\nu }_{\eta }$. Contracting this result with $g^{\alpha \eta }$, together with relation $U^{\nu }\nabla _{\nu }U^{\mu }=d^2x^{\mu }/ds^2+\Gamma ^{\mu }_{\nu \eta }U^{\nu }U^{\eta }$, leads to the equation of motion for massive test particles in context of the $f\left( \mathcal {G},\phi ,(\nabla \phi )^2,T\right)$ gravity:

23 $$\begin{aligned} \frac{d^2x^{\mu }}{ds^2}+\Gamma ^{\mu }_{\nu \eta }U^{\nu }U^{\eta }=\zeta ^{\mu }. \end{aligned}$$

The test particles will experience extra force $\zeta ^{\mu }$:

24 $$\begin{aligned} \zeta ^{\mu }=\frac{(2\chi +f_T(\mathcal {G},\phi ,(\nabla \phi )^2,T))}{(\rho +P)(2\chi +3f_T(\mathcal {G},\phi ,(\nabla \phi )^2,T)}\left( \nabla ^{\mu }P-U^{\mu }U^{\nu }\nabla _{\nu }P\right) , \end{aligned}$$

and follow nongeodesic trajectories. This force is the direct consequence of non-minimal coupling between geometry and matter, a common property of theories with such couplings^6,22,23^. In absence of the matter–geometry coupling, motion of the test particles will be the same as in the general relativity, as a consequence of the extra force absence ($\zeta ^\mu =0$).

The FLRW (Friedmann–Lemaître–Robertson–Walker) line element is given as:

25 $$\begin{aligned} ds^2=dt^2-a^2(t)(dx^2+dy^2+dz^2), \end{aligned}$$

where *a*(*t*) is the scale factor depending on cosmic time *t*. We remark, that the metric given above describes homogeneous and isotropic Universe^55,77^. For the FLRW spacetime, scalar field will depend only on time coordinate *t* i.e. $\phi =\phi (t)$. Hence, from Eq. (10) the corresponding field equations for the 00th and *ii*th components are:

26 $$\begin{aligned} 3H^2=\chi \rho _{eff},\; -(2\dot{H}+3H^2)=\chi P_{eff}, \end{aligned}$$

with

27 $$\begin{aligned} \rho _{eff}&=\rho +\frac{1}{\chi }\left[ (\rho +P)f_T+\frac{1}{2}f-12H^2(\dot{H}+H^2)f_\mathcal {G}+12H^3\partial _tf_\mathcal {G}-\dot{\phi }^2f_{(\nabla \phi )^2}\right] , \end{aligned}$$

28 $$\begin{aligned} P_{eff}&=P-\frac{1}{\chi }\left[ \frac{1}{2}f-12H^2(\dot{H}+H^2)f_\mathcal {G}+8H(\dot{H}+H^2)\partial _tf_\mathcal {G}+4H^2\partial _{tt}f_\mathcal {G} \right] , \end{aligned}$$

where overdot indicates derivative with respect to *t*, and *H* is the Hubble parameter defined as $H=\dot{a}(t)/a(t)$. Ricci scalar and the GB term are equal to:

29 $$\begin{aligned} R=-6(\dot{H}+2H^2), \; \mathcal {G}=24H^2(\dot{H}+H^2). \end{aligned}$$

The corresponding scalar equation takes form:

30 $$\begin{aligned} \frac{1}{2}f_\phi =\partial _tf_{(\nabla \phi )^2}\dot{\phi }+f_{(\nabla \phi )^2}(\ddot{\phi }+3H\dot{\phi }). \end{aligned}$$

Furthermore, kinetic term is equal to: $(\nabla \phi )^2=g^{\mu \nu }\nabla _\mu \phi \nabla _\nu \phi =\dot{\phi }^2$. Covariant divergence of $T_{\mu \nu }$ for the FLRW metric obtained from Eq. (13) is given by:

31 $$\begin{aligned} \dot{\rho }+3H(\rho +P)=-\frac{1}{\chi +f_T}\left[ \left( \frac{1}{2}\dot{T}+P\right) f_T+(\rho +P)\partial _tf_T\right] . \end{aligned}$$

To restore usual conservation equation we need to set both sides of this equation to zero. This results in the conservation equation:

32 $$\begin{aligned} \dot{\rho }+3H(\rho +P)=0, \end{aligned}$$

together with constraint:

33 $$\begin{aligned} \left( \frac{1}{2}\dot{T}+P\right) f_T+(\rho +P)\partial _tf_T=0. \end{aligned}$$

## Energy conditions

Energy conditions are important tool in the GR and are often used in case of the modified gravity theories^68–71^. In the GR context, they can help in choosing physically reasonable matter contents^54^. They were also used in proving laws of thermodynamics and various black hole theorems^56^. In context of the discussed theory, the energy conditions may be used to constraint parameters and check validity of (effective) matter content in the cosmological scenarios. The starting point of energy conditions are the Raychaudhuri equations, describing congruences of geodesics on the manifold. These equations are used in discussing gravity as an attractive force and the positiveness of matter energy density by the means of evolution of expansion scalar ($\theta$). Moreover, extensions of the GR should also be challenged with the energy conditions, since they assign geodesic and causal structure of the spacetime manifold. The Raychaudhuri equations are given by^54^:

34 $$\begin{aligned} \frac{d\theta }{d\tau }&=-\frac{1}{3}\theta ^2+\omega _{\mu \nu }\omega ^{\mu \nu }-\sigma _{\mu \nu }\sigma ^{\mu \nu }-R_{\mu \nu }u^\mu u^\nu , \end{aligned}$$

35 $$\begin{aligned} \frac{d\theta }{d\tau }&=-\frac{1}{2}\theta ^2+\omega _{\mu \nu }\omega ^{\mu \nu }-\sigma _{\mu \nu }\sigma ^{\mu \nu }-R_{\mu \nu }k^\mu k^\nu , \end{aligned}$$

where $\sigma _{\mu \nu }$, $\omega _{\mu \nu }$ are shear tensor and rotation; the timelike and null tangent vectors are denoted as $u^{\mu }$ and $k^{\mu }$. Neglecting small distortions and second order terms, from the Raychaudhuri equation one gets:

36 $$\begin{aligned} \theta =-\tau R_{\mu \nu }u^\mu u^\nu ,\; \theta =-\tau R_{\mu \nu }k^\mu k^\nu . \end{aligned}$$

Condition for the attractive gravity (SEC), i.e. $\theta <0$, gives inequalities:

37 $$\begin{aligned} R_{\mu \nu }u^\mu u^\nu&=\left( T_{\mu \nu }-\frac{1}{2}g_{\mu \nu }T\right) u^\mu u^\nu \ge 0,\nonumber \\ R_{\mu \nu }k^\mu k^\nu&=\left( T_{\mu \nu }-\frac{1}{2}g_{\mu \nu }T\right) k^\mu k^\nu \ge 0, \end{aligned}$$

where we have used combination of the energy–momentum tensor and its trace. Inequalities (37) provide energy conditions for a perfect fluid:

- NEC: $$\rho +P\ge 0$$,
- WEC: $$\rho \ge 0$$, $$\rho +P\ge 0$$,
- SEC: $$\rho +P\ge 0$$, $$\rho +3P\ge 0$$,
- DEC: $$\rho \ge 0$$, $$\rho \pm P\ge 0$$,

that obey:

38 $$\begin{aligned} DEC \Longrightarrow WEC \Longrightarrow NEC \Longleftarrow SEC. \end{aligned}$$

Thus, violation of NEC leads to the violation of other listed conditions.

For the modified theories of gravity, energy constraints can be extended, due to the geometric character of the Raychaudhuri equations. We note that this is somewhat problematic in the case of the modified gravities, where it is not exactly clear which part of gravity enters the effective matter tensor. Moreover, the energy conditions in the extended theories of gravity emerge not only from $T_{\mu \nu }$ but also from the geometrical quantity $T^{grav}_{\mu \nu }$. It means that standard GR interpretation of the energy conditions may not be the same in the modified theories of gravity, as the additional fluids coming from the corrections in the effective tensor ($T_{\mu \nu }^{eff}$) may carry different physical properties than the standard matter fluid^64^. For example, in the *f*(*R*) theory, when SEC is valid, one may obtain repulsive gravity^75,76^. Moreover, the energy conditions satisfied in one of the conformal related frames, may not be satisfied in another conformal frame. As an example, NEC validity in the Jordan frame does not necessarily imply validity in the Einstein frame^63^. For more detailed discussion on the interpretation of the energy conditions and effective energy–momentum tensor, we refer reader to the detailed studies^63,64,75,76^.

We can assume that matter distribution acts like a perfect fluid. The translated energy conditions for the effective fluid described by the energy–momentum tensor $T^{eff}_{\mu \nu }=diag(\rho _{eff},P_{(eff)i})$ in $f\left( \mathcal {G},\phi ,(\nabla \phi )^2,T\right)$, are ($P=0$):

- NEC:
  39 $$\begin{aligned} \rho _{eff}+P_{eff}=\rho + \frac{1}{\chi }\left[ \rho f_T+4H(2\dot{H}-H^2) \partial _t f_{\mathcal {G}} -4H^2\partial _{tt} f_{\mathcal {G}} -\dot{\phi }^2 f_{(\nabla \phi )^2} \right] \ge 0, \end{aligned}$$
- WEC:
  40 $$\begin{aligned} \rho _{eff}&=\rho +\frac{1}{\chi }\left[ \rho f_T+\frac{1}{2}f-12H^2(\dot{H}+H^2)f_\mathcal {G}+12H^3\partial _tf_\mathcal {G}-\dot{\phi }^2f_{(\nabla \phi )^2}\right] \ge 0, \end{aligned}$$
- SEC:
  41 $$\begin{aligned} \rho _{eff}+3P_{eff}=\rho -\frac{1}{\chi }\left[ f-\rho f_T -24H^2(\dot{H}+H)f_{\mathcal {G}} +12H^2(2\dot{H}+H)\partial _t f_{\mathcal {G}}+12H^2\partial _{tt}f_{\mathcal {G}} + \dot{\phi }^2 f_{(\nabla \phi )^2} \right] \ge 0, \end{aligned}$$
- DEC:
  42 $$\begin{aligned} \rho _{eff}-P_{eff}=\rho +\frac{1}{\chi }\left[ \rho f_T+f -24H^2(\dot{H}+H^2)f_{\mathcal {G}}+4H(2\dot{H}+5H^2)\partial _t f_{\mathcal {G}}+4H^2\partial _{tt}f_{\mathcal {G}} -\dot{\phi }^2f_{(\nabla \phi )^2}\right] \ge 0. \end{aligned}$$

We remark, that it is possible to obtain energy conditions of the $f(\mathcal {G},T)$, or the $f(\mathcal {G})$ theories by choosing the $f\left( \mathcal {G},\phi ,(\nabla \phi )^2,T\right) \rightarrow f(\mathcal {G})$, or $\rightarrow f(\mathcal {G},T)$ in the action, instead of the $f\left( \mathcal {G},\phi ,(\nabla \phi )^2,T\right)$ approach. Before we move on, it is worth noting that the chain rule for the second derivative of function $\partial _{tt}f_{\mathcal {G}}\left( \mathcal {G},\phi ,(\nabla \phi )^2,T\right)$ can be expanded as:

43 $$\begin{aligned} \partial _{tt}f_{\mathcal {G}}&= 2\left( \dot{\mathcal {G}}\dot{\phi }f_{\mathcal {G}\mathcal {G}\phi } + \dot{\mathcal {G}}\dot{X}f_{\mathcal {G}\mathcal {G}X} +\dot{\mathcal {G}}\dot{T}f_{\mathcal {G}\mathcal {G}T}+\dot{\phi }\dot{X}f_{\mathcal {G}\phi X}+ \dot{\phi }\dot{T}f_{\mathcal {G}\phi T}+\dot{X}\dot{T}f_{\mathcal {G}X T}\right) \nonumber \\&\quad + \ddot{\mathcal {G}}f_{\mathcal {G}\mathcal {G}}+\ddot{\phi }f_{\mathcal {G}\phi }+\ddot{X}f_{\mathcal {G}X}+\ddot{T}f_{\mathcal {G}T}+\dot{\mathcal {G}}^2f_{\mathcal {G}\mathcal {G}\mathcal {G}}+\dot{\phi }^2f_{\mathcal {G}\phi \phi }+\dot{X}^2f_{\mathcal {G}XX}+\dot{T}^2f_{\mathcal {G}TT}, \end{aligned}$$

where for simplicity kinetic term is denoted as $X=(\nabla \phi )^2$. Sometimes it is worth to introduce dimensionless cosmological parameters, decceleration (*q*), jerk (*j*) and snap (*s*)^78,79^:

44 $$\begin{aligned} q=-\frac{1}{H^2}\frac{\ddot{a}}{a}, \;j=\frac{1}{H^3}\frac{\dddot{a}}{a}, \;s=\frac{1}{H^4}\frac{\ddddot{a}}{a}. \end{aligned}$$

The present-day value of jerk describes different dark energy (DE) models causing acceleration of the universe, while negative value of deceleration, together with current Hubble parameter describes rate of expansion for accelerating universe. Using these parameters, the Hubble parameter, as well as the Ricci and GB scalars can be written as:

45 $$\begin{aligned} \dot{H}&=-H^2(1+q),\;\ddot{H}=H^3(j+3q+2), \; \dddot{H}=H^4(s-4j-3q^2-12q-6), \nonumber \\ R&=-6H^2(1-q), \; \dot{R}=-6H^3(j-q-2),\; \ddot{R}=-6H^4(s+8q+q^2+6), \nonumber \\ \mathcal {G}&=-24qH^4,\; \dot{\mathcal {G}}=24H^5(j+2q^2+3q),\; \ddot{\mathcal {G}}=24H^6(s-6j-6jq-12q-15q^2-2q^3). \end{aligned}$$

Thus, energy conditions rewritten with aid of the above expressions are:

- NEC:
  46 $$\begin{aligned} \rho _{eff}+P_{eff}&=\rho + \frac{1}{\chi }\left[ \rho f_T+4H^3(3+2q) ({\mathcal {G}}f_{\mathcal {G}\mathcal {G}}+ \dot{\phi } f_{\mathcal {G}\phi }+\dot{X} f_{\mathcal {G}X}+\dot{T} f_{\mathcal {G}T} )-X f_{X}\right. \nonumber \\&\quad -4H^2\left( 2( \dot{\mathcal {G}}\dot{\phi }f_{\mathcal {G}\mathcal {G}\phi } + \dot{\mathcal {G}}\dot{X}f_{\mathcal {G}\mathcal {G}X} +\dot{\mathcal {G}}\dot{T}f_{\mathcal {G}\mathcal {G}T} +\dot{\phi }\dot{X}f_{\mathcal {G}\phi X}+ \dot{\phi }\dot{T}f_{\mathcal {G}\phi T}+\dot{X}\dot{T}f_{\mathcal {G}X T})\right. \nonumber \\&\quad \left. \left. + \ddot{\mathcal {G}}f_{\mathcal {G}\mathcal {G}}+\ddot{\phi }f_{\mathcal {G}\phi }+\ddot{X}f_{\mathcal {G}X}+\ddot{T}f_{\mathcal {G}T}+\dot{\mathcal {G}}^2f_{\mathcal {G}\mathcal {G}\mathcal {G}}+\dot{\phi }^2f_{\mathcal {G}\phi \phi }+\dot{X}^2f_{\mathcal {G}XX}+\dot{T}^2f_{\mathcal {G}TT}\right) \right] \ge 0, \end{aligned}$$
- WEC:
  47 $$\begin{aligned} \rho _{eff}=\rho +\frac{1}{2\chi }\left[ 2\rho f_T+f+24qH^4f_\mathcal {G}+24H^3(\dot{\mathcal {G}}f_{\mathcal {G}\mathcal {G}}+ \dot{\phi } f_{\mathcal {G}\phi }+\dot{X} f_{\mathcal {G}X}+\dot{T} f_{\mathcal {G}T} )-2Xf_{X}\right] \ge 0, \end{aligned}$$
- SEC:
  48 $$\begin{aligned} \rho _{eff}+3P_{eff}&=\rho +\frac{1}{\chi }\left[ -f+\rho f_T -24qH^4f_\mathcal {G}+12H^3(1+2q)(\dot{\mathcal {G}}f_{\mathcal {G}\mathcal {G}}+ \dot{\phi } f_{\mathcal {G}\phi }+\dot{X} f_{\mathcal {G}X}+\dot{T} f_{\mathcal {G}T} ) f_{\mathcal {G}}- X f_{X}\right. \nonumber \\&\quad -12H^2\left( 2( \dot{\mathcal {G}}\dot{\phi }f_{\mathcal {G}\mathcal {G}\phi } + \dot{\mathcal {G}}\dot{X}f_{\mathcal {G}\mathcal {G}X} +\dot{\mathcal {G}}\dot{T}f_{\mathcal {G}\mathcal {G}T}+\dot{\phi }\dot{X}f_{\mathcal {G}\phi X}+ \dot{\phi }\dot{T}f_{\mathcal {G}\phi T}+\dot{X}\dot{T}f_{\mathcal {G}X T})\right. \nonumber \\&\quad \left. \left. + \ddot{\mathcal {G}}f_{\mathcal {G}\mathcal {G}}+\ddot{\phi }f_{\mathcal {G}\phi }+\ddot{X}f_{\mathcal {G}X}+\ddot{T}f_{\mathcal {G}T}+\dot{\mathcal {G}}^2f_{\mathcal {G}\mathcal {G}\mathcal {G}}+\dot{\phi }^2f_{\mathcal {G}\phi \phi }+\dot{X}^2f_{\mathcal {G}XX}+\dot{T}^2f_{\mathcal {G}TT}\right) \right] \ge 0, \end{aligned}$$
- DEC:
  49 $$\begin{aligned} \rho _{eff}-P_{eff}&=\rho +\frac{1}{\chi }\left[ \rho f_T +f -X f_{X} +24qH^4f_{\mathcal {G}}+4H^3(3-2q)\left( \dot{\mathcal {G}}f_{\mathcal {G}\mathcal {G}}+ \dot{\phi } f_{\mathcal {G}\phi }+\dot{X} f_{\mathcal {G}X}+\dot{T} f_{\mathcal {G}T} \right) \right. \nonumber \\&\quad +4H^2\left( 2\left( \dot{\mathcal {G}}\dot{\phi }f_{\mathcal {G}\mathcal {G}\phi } + \dot{\mathcal {G}}\dot{X}f_{\mathcal {G}\mathcal {G}X} +\dot{\mathcal {G}}\dot{T}f_{\mathcal {G}\mathcal {G}T}+\dot{\phi }\dot{X}f_{\mathcal {G}\phi X}+ \dot{\phi }\dot{T}f_{\mathcal {G}\phi T}+\dot{X}\dot{T}f_{\mathcal {G}X T}\right) \right. \nonumber \\&\quad \left. \left. + \ddot{\mathcal {G}}f_{\mathcal {G}\mathcal {G}}+\ddot{\phi }f_{\mathcal {G}\phi }+\ddot{X}f_{\mathcal {G}X}+\ddot{T}f_{\mathcal {G}T}+\dot{\mathcal {G}}^2f_{\mathcal {G}\mathcal {G}\mathcal {G}}+\dot{\phi }^2f_{\mathcal {G}\phi \phi }+\dot{X}^2f_{\mathcal {G}XX}+\dot{T}^2f_{\mathcal {G}TT}\right) \right] \ge 0. \end{aligned}$$

## Cosmological reconstruction

The main purpose of introducing Einstein’s gravity extensions is to study cosmological solutions coming from a given theory. However, cosmological equations from postulated theories are often hard to solve, even with large number of assumptions and simplifications. In the so called reconstruction techniques, known cosmological solution is given and equations are solved for particular model coming from the theory, which satisfy given cosmological evolution (i.e. spacetime is *reconstructed* in the gravitational theory of interest)^20,68,80–85^. Such reconstructed models can be further studied and compared with experimental data, in order to check physical importance and viability. In this study we obtain models satisfying the de-Sitter and power-law evolution.

As a brief note, in a given reconstruction procedure it is possible to reconstruct any given cosmology, once the scale factor is specified. For example, using proper scale factor with the form $a(t)=a_0 exp(g(t))$ (thus $H=\dot{g}(t)$) one can obtain proper function $f(G,\phi ,(\nabla \phi )^2,T)$ with the scalar field redefined as $\phi =t$. To unify matter-dominated and accelerated phases, one can choose $g(t)=h(t)ln(t)$ with $h=(h_1+h_2t^2)/(1+qt^2)$ where $h_1$, $h_2$ and *q* are constants^81^. Then, choosing appropriate *h* with adiabatic approximation, one can reconstruct specific model describing transition between phases and investigate its asymptotic form, for small ($t\rightarrow 0$) or large ($t\rightarrow \infty$) curvature. Similarly, one can unify inflation with recent acceleration for $H(t)=H_0 \frac{1+\epsilon (t/t_0)^2}{1+(t/t_0)^2}$^80^. This procedure can also be extended to other cosmological models (such as the Bounce or $\Lambda$CDM approach). Thus, using reconstruction techniques it is possible to recover the whole Universe history. For extensive discussion on the reconstruction procedure in the modifications of the GR, we refer reader to notable review^10^.

### de-Sitter spacetime

The de-Sitter model describes exponential growth of universe and is characterised by the constant Hubble parameter and the constant curvature. The scale factor and the Hubble parameter are given by:

50 $$\begin{aligned} a(t)=a_0 e^{H_0t}, \; H=H_0, \end{aligned}$$

where $a_0$ is constant corresponding to $t=t_0$. The Ricci and GB curvature scalars are:

51 $$\begin{aligned} R=-12H_0^2,\; \mathcal {G}=24H_0^4. \end{aligned}$$

For scalar field $\phi$ we use^86,87^:

52 $$\begin{aligned} \phi (t)\sim a(t)^\beta =a_0^\beta e^{\beta H_0t}, \end{aligned}$$

together with corresponding derivative:

53 $$\begin{aligned} \dot{\phi }= \beta H_0 \phi . \end{aligned}$$

Using Eq. (32) for pressureless ($P=0$) fluid one can obtain energy density:

54 $$\begin{aligned} \rho =\rho _0e^{-3H_0t}, \end{aligned}$$

provided with the trace of the energy–momentum tensor and its derivatives:

55 $$\begin{aligned} T=\rho , \; \dot{T}=-3 H_0 T, \; \ddot{T}=9H_0^2 T. \end{aligned}$$

We can greatly simplify our considerations by using specific case, i.e. $f\left( \mathcal {G},\phi ,X,T\right) =\mathcal {E}(\mathcal {G},\phi ,T)+\omega (\phi )X$. We note, that idea of coupling between geometry, scalar field and matter is still present and our considerations become simplified. Moreover $\omega (\phi )$ is natural choice of the kinetic term coupling in the scalar tensor theories^32^. Using power form of $\omega$^68,86,87^:

56 $$\begin{aligned} \omega (\phi )= \omega _0\phi ^m. \end{aligned}$$

Then, from first of the Eqs. (26) and (27) we have:

57 $$\begin{aligned} \chi T+\frac{1}{2}(\mathcal {E}-\omega _0\beta ^2 H_0^2\phi ^{m+2})+T\mathcal {E}_T-12H_0^4\mathcal {E}_\mathcal {G}+12\beta H_0^4\phi \mathcal {E}_{\mathcal {G}\phi }-36H_0^4T\mathcal {E}_{\mathcal {G}T}-3H_0^2=0, \end{aligned}$$

with solution:

58 $$\begin{aligned} \mathcal {E}(\mathcal {G},\phi ,T)=C_1C_2C_3\left[ e^{C_1\mathcal {G}}\phi ^{C_2}T^{d_1}+T^{d_2}\right] +d_3+d_4T+d_5 \phi ^{d_6}, \end{aligned}$$

where:

59 $$\begin{aligned} d_1=\frac{1}{2}\left[ \frac{24(\beta C_2-1)H_0^4C_1+1}{36H_0^4C_1-1}\right] , \; d_2=-\frac{1}{2},\; d_3=6H_0, \; d_4=- \frac{2}{3}\chi , \; d_5=\omega _0 \beta ^2 H_0^2, \; d_6=m+2. \end{aligned}$$

Energy conditions take the form:

- NEC
  60 $$\begin{aligned} \rho _{eff}+P_{eff}&=\rho +\frac{1}{\chi }\left[ \rho (C_1C_2C_3(d_1e^{C_1\mathcal {G}}\phi ^{C_2}T^{d_1}+d_2T^{d_2-1})+d_4)+4H_0^3(3+2q)\left\{ \beta H_0 C_1^1C_2^2C_3 e^{C_1\mathcal {G}}\phi ^{C_2}T^{d_1} \right. \right. \nonumber \\&\quad \left. -3H_0C_1^2C_2C_3d_1e^{C_1\mathcal {G}}\phi ^{C_2}T^{d_1} \right\} -4 H^2 \left\{ \beta ^2 C_1^2 C_2^2 C_3 H^2 e^{C_1 \mathcal {G}} \phi ^{C_2} T^{d_1}+\beta ^2 C_1^2 (C_2-1) C_2^2 C_3 H^2 e^{C_1 \mathcal {G}} \phi ^{C_2} T^{d_1}\right. \nonumber \\&\quad \left. \left. -6 \beta C_1^2 C_2^2 C_3 d_1 H^2 e^{C_1 \mathcal {G}} \phi ^{C_2} T^{d_1}+9 C_1^2 C_2 C_3 d_1 H^2 e^{C_1 \mathcal {G}} \phi ^{C_2} T^{d_1}\right\} -d_5 \phi ^{d_6}\right] , \end{aligned}$$
- WEC
  61 $$\begin{aligned} \rho _{eff}&=\rho +\frac{1}{2\chi }\left[ 24 C_1^2 C_2 C_3 H^4 q e^{C_1 \mathcal {G}} \phi ^{C_2} T^{d_1}+24 H^3 \left( \beta C_1^2 C_2^2 C_3 H e^{C_1 \mathcal {G}} \phi ^{C_2} T^{d_1}-3 C_1^2 C_2 C_3 d_1 H e^{C_1 \mathcal {G}} \phi ^{C_2} T^{d_1}\right) \right. \nonumber \\&\quad \left. +2 \rho \left( C_1 C_2 C_3 d_1 e^{C_1 \mathcal {G}} \phi ^{C_2} T^{d_1-1}+C_1 C_2 C_3 d_2 T^{d_2-1}+d_4\right) +C_1 C_2 C_3 e^{C_1 \mathcal {G}} \phi ^{C_2} T^{d_1}+C_1 C_2 C_3 T^{d_2}+d_4 T+d_3 \right] . \end{aligned}$$

In this case, we have six parameters ($C_1,C_2,C_3,m,\beta ,t$), thus graphical description of the NEC and WEC validity is possible when three of the parameters are fixed. For simplicity we set $m=1$ for all the considered choices of other parameters. We note that we set $a_0=\phi _0=\omega _0=1$. We use following values of the Hubble parameter and the cosmographical parameters: $H_0=0.718$, $q=-0.64$, $j=-1.02$, $s=-0.39$^20,66,88^.

**Figure 1:**
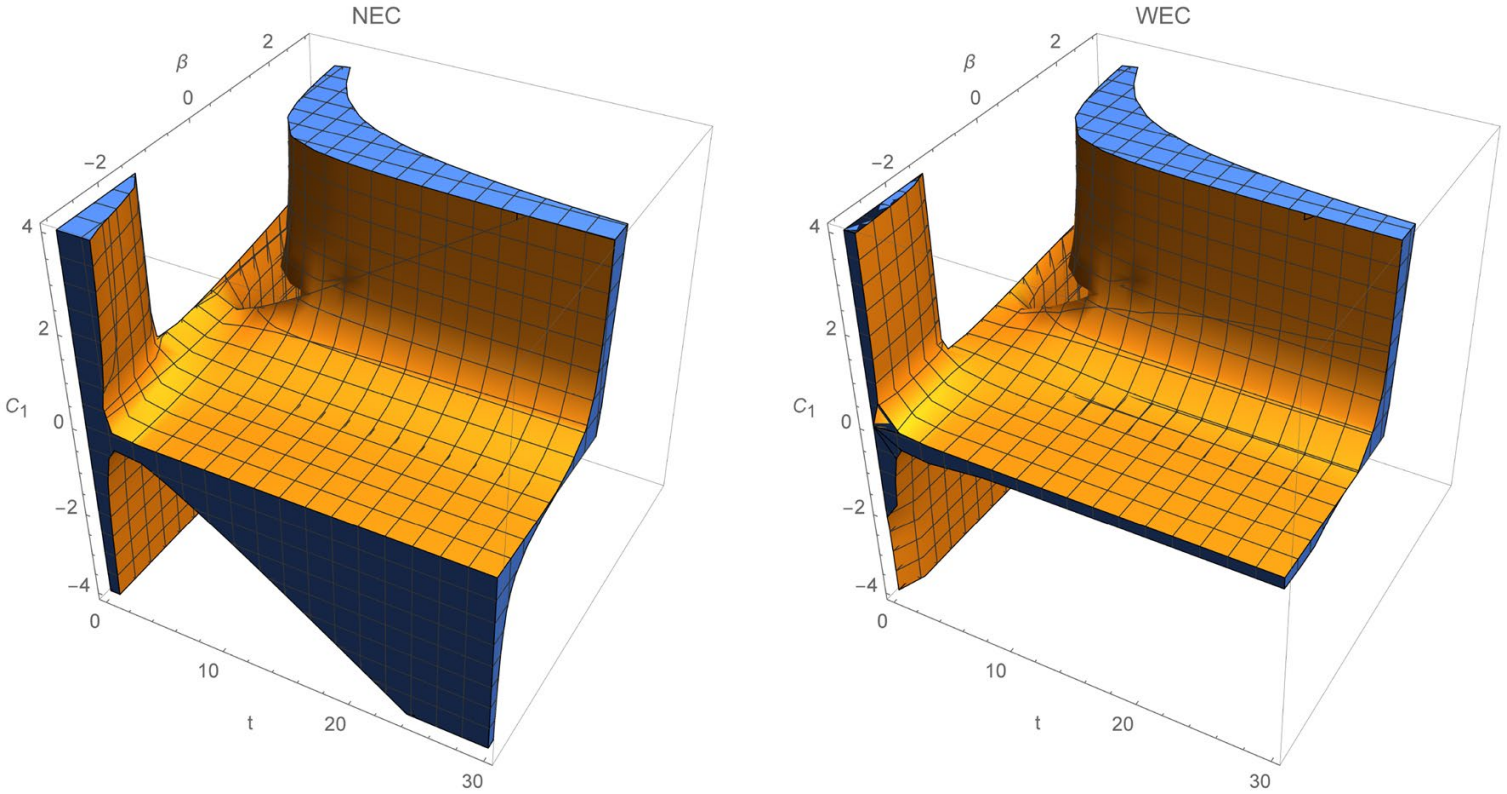
Regions where NEC and WEC are valid for $C_2=-0.6$.

In the first case, we fix integration constants $C_3=0.5$ and $C_2$. Figure 1 show regions where NEC and WEC hold, when $C_2=-0.6$. For all given time intervals $t\in (0,30)$, NEC and WEC will hold for any constant range $\beta \in (-4,4)$ for $C_1\sim 0$, while for $C_1<0$ the energy conditions will be true for all $\beta$ parameters.

**Figure 2:**
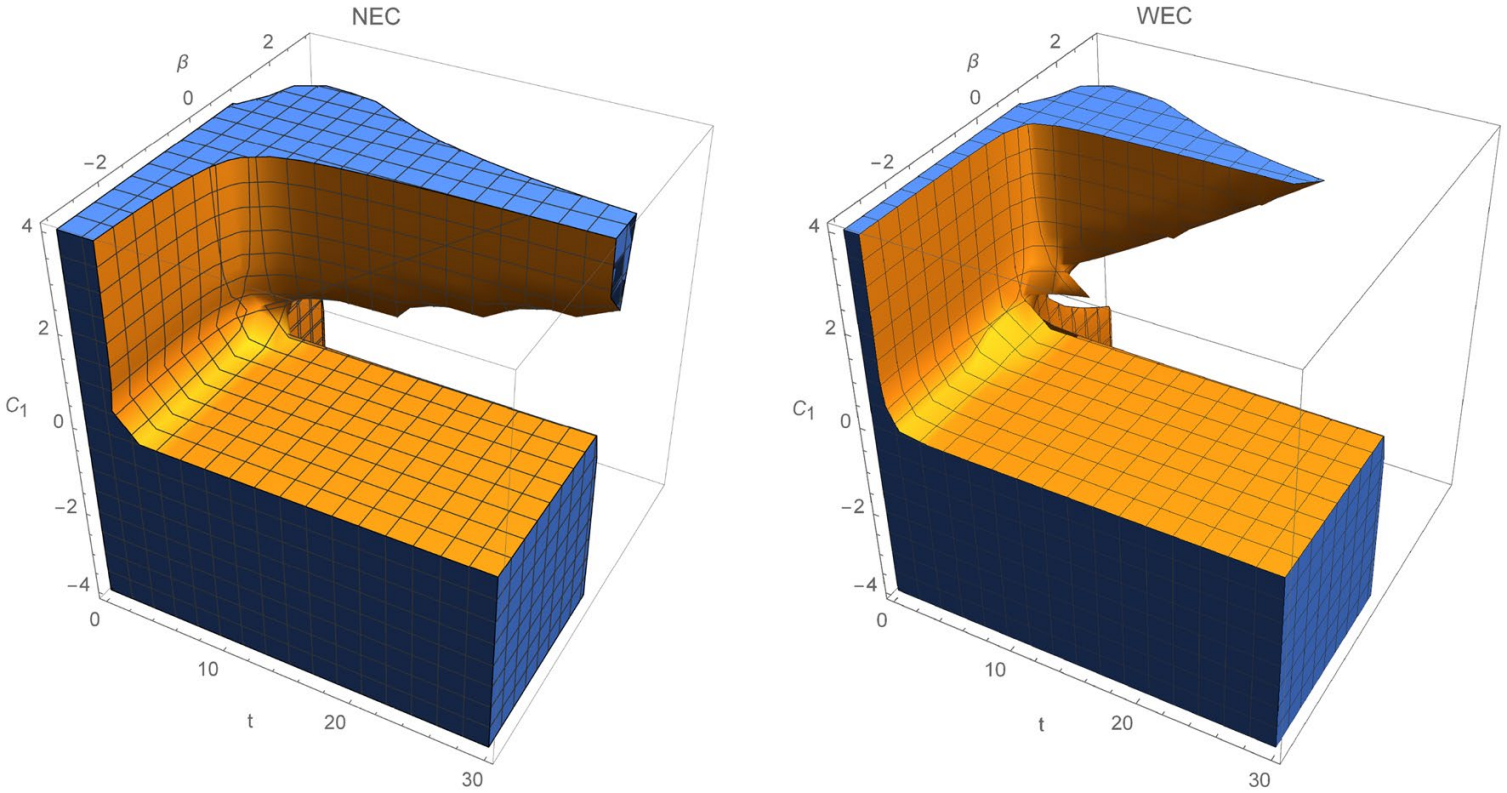
Regions where NEC and WEC are valid for $C_2=1$.

**Figure 3:**
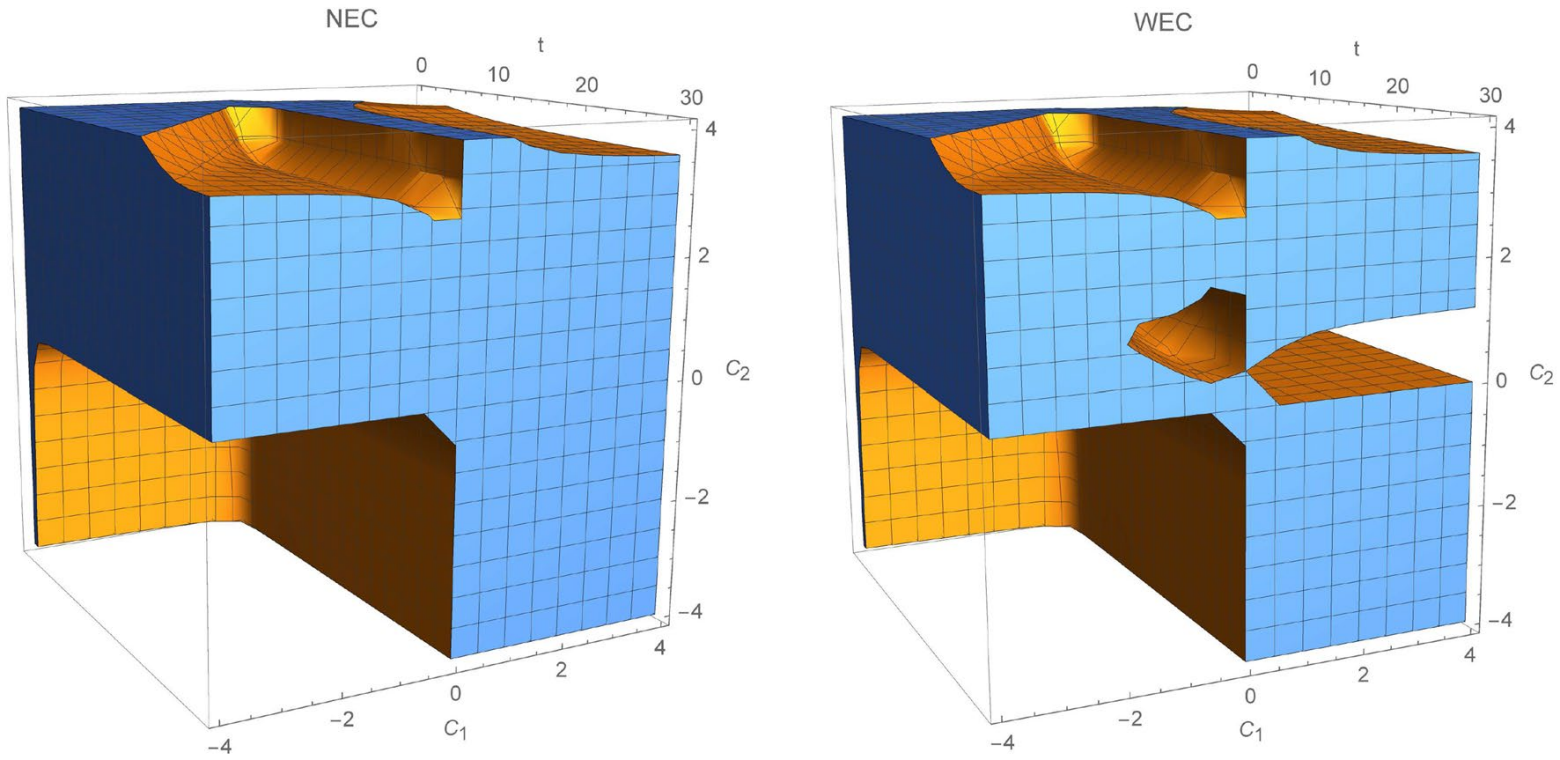
Validity of NEC and WEC with respect to time *t* and constants $C_1$,$C_2$ where $\beta =0.5$.

In Fig. 2, the validity of energy conditions is described for specific choice of $C_2>0$. NEC and WEC are fulfilled at any given time when $\beta ,C_1<0$. Clearly, region where WEC holds is much smaller than for NEC, since NEC is a weaker condition than WEC [from relation (38)]. In Fig. 3, NEC and WEC are presented. In this case, we set $\beta =0.5$ and let constants $C_1$ and $C_2$ run through $(-4,4)$. Regions where WEC and NEC are valid are very similar, except region where $C_1>0.8\wedge C_2 \in (0,-1.4)$.

### Power law solutions

Power law models are important in discussing cosmic evolution and epochs. Their scale factor, the Hubble parameter and the energy density are given by:

62 $$\begin{aligned} a(t)=a_0 t^n, \; H=\frac{n}{t}, \; \rho =\rho _0 t^{-3n}, \end{aligned}$$

where $n>0$. For $n>1$, accelerated expansion of the universe occurs, while for the deccelerated phase we have $1>n>0$, which leads to the radiation ($n=1/2$) or dust ($n=2/3$) dominated universe. Corresponding energy momentum tensor and its time derivatives are given by:

63 $$\begin{aligned} T=\rho , \, \; \dot{T}=-\frac{3n}{t}T, \, \; \ddot{T}=\frac{3n}{t^2}(3n+1)T. \end{aligned}$$

The Ricci scalar and Gauss–Bonnet term are equal to:

64 $$\begin{aligned} R=\frac{6n}{t^2}(1-2n), \; \mathcal {G}=24\frac{n^3}{t^4}(n-1). \end{aligned}$$

Similarly to the de-Sitter model, we again consider fixed kinetic term. The scalar field and its derivative takes form:

65 $$\begin{aligned} \phi (t)\sim a(t)^\beta =a_0^\beta t^{\beta n},\; \dot{\phi }(t)=\frac{\beta n}{t}\phi . \end{aligned}$$

Hence, for the power-law scale factor and again $f\left( \mathcal {G},\phi ,X,T\right) =\mathcal {E}(\mathcal {G},\phi ,T)+\omega (\phi )X$, Eqs. (26) and (27) take form:

66 $$\begin{aligned}&\chi T+ T\mathcal {E}_T-\frac{1}{2}\mathcal {G}\mathcal {E}_\mathcal {G}+\frac{1}{2}(\mathcal {E}-\omega _0 \beta ^2 n^2 a_0 ^{\frac{2}{n}}\phi ^{m+2-\frac{2}{\beta n}}) -\left( \frac{2}{n-1}\right) \mathcal {G}^2 \mathcal {E}_{\mathcal {G}\mathcal {G}}-\left( \frac{3n}{2(n-1)}\right) \mathcal {G}T\mathcal {E}_{\mathcal {G}T} +\left( \frac{\beta n}{2(n-1)}\right) \mathcal {G}\phi \mathcal {E}_{\mathcal {G}\phi }\nonumber \\&\quad -3n^2\left( \frac{T}{\rho _0}\right) ^{\frac{2}{3n}} =0. \end{aligned}$$

In what follows, the solution of this partial differential equation is:

67 $$\begin{aligned} \mathcal {E}(\mathcal {G},\phi ,T)=C_1C_3C_4\phi ^{C_2}T^{C_3}\mathcal {G}^{\frac{1}{4}(d_1+d_2)}+C_1C_2C_3 T^{d_3} +C_2C_3C_4 \phi ^{C_2}T^{C_3}\mathcal {G}^{\frac{1}{4}(d_1-d_2)}+d_4T^{d_5}+d_6T +d_7 \phi ^{d_8}, \end{aligned}$$

where:

68 $$\begin{aligned}&d_1=\frac{1}{2}[C_2\beta n-(3C_3+1)n+5]\nonumber \\&d_2=\frac{1}{2}\left[ {n}^{2}{C_{{2}}}^{2}-6C_2n\beta \left( ( C_{{3}}+\frac{1}{3} ) n -\frac{5}{3}\right) +9{n}^{2} ( C_{{3}}+ \frac{1}{3} ) ^{2}+ n( 2C_{{3}}+6 ) -32C_{{3}}+9\right] \nonumber \\&d_3=-\frac{1}{2}, \; d_4=\left( \frac{18n^3}{3n+4}\right) \rho _0^{-\frac{2}{3n}},\; \;d_5=\frac{2}{3n},\; d_6=-\frac{2}{3}\chi ,\; d_7=\omega _0 \beta ^2n^2a_0^{\frac{2}{n}}, \; d_8=m+2-\frac{2}{\beta n}. \end{aligned}$$

From this solution and from Eqs. (39) and (40) the energy conditions are:

- NEC
  69 $$\begin{aligned} \rho _{eff}+P_{eff}&=\rho + \frac{1}{\chi }\left[ \rho (C_3 C_4 (C_1 C_3 T^{C_3-1} \phi ^{C_2} \mathcal {G}^{\frac{1}{4} (d_1+d_2)}+C_2 C_3 T^{C_3-1} \phi ^{C_2} \mathcal {G}^{\frac{1}{4} (d_1-d_2)})+C_1 C_2 C_3 d_3 T^{d_3-1}\right. \nonumber \\&\quad +d_4 d_5 T^{d_5-1}+d_6)-12 H^3 (2 q+1) \{C_3 C_4 \dot{T} (\frac{1}{4} C_2 C_3 (d_1-d_2) T^{C_3-1} \phi ^{C_2} \mathcal {G}^{\frac{1}{4} (d_1-d_2)-1}+\frac{1}{4} C_1 C_3 (d_1+d_2) T^{C_3-1} \phi ^{C_2}\nonumber \\&\quad \times \mathcal {G}^{\frac{1}{4} (d_1+d_2)-1})+C_3 C_4 \dot{\phi } (\frac{1}{4} C_2^2 (d_1-d_2) T^{C_3} \phi ^{C_2-1} \mathcal {G}^{\frac{1}{4} (d_1-d_2)-1}+\frac{1}{4} C_1 C_2 (d_1+d_2) T^{C_3} \phi ^{C_2-1} \mathcal {G}^{\frac{1}{4} (d_1+d_2)-1})+C_3 C_4 \dot{\mathcal {G}}\nonumber \\&\quad \times (\frac{1}{4} C_2 (\frac{1}{4} (d_1-d_2)-1) (d_1-d_2) T^{C_3} \phi ^{C_2} \mathcal {G}^{\frac{1}{4} (d_1-d_2)-2}+\frac{1}{4} C_1 (d_1+d_2) (\frac{1}{4} (d_1+d_2)-1) T^{C_3} \phi ^{C_2} \mathcal {G}^{\frac{1}{4} (d_1+d_2)-2})\Big \} -4 H^2 \nonumber \\&\quad \times \left\{ C_3 C_4 (\frac{1}{4} T^{C_3-2} \mathcal {G}^{\frac{1}{4} (d_1-d_2)-1} C_2 (C_3-1) C_3 (d_1-d_2) \phi ^{C_2}+\frac{1}{4} T^{C_3-2} \mathcal {G}^{\frac{1}{4} (d_1+d_2)-1} C_1 (C_3-1) C_3 (d_1+d_2) \phi ^{C_2}) \dot{\mathcal {G}}^2 \right. \nonumber \\&\quad +C_3 C_4 (\frac{1}{4} T^{C_3} \mathcal {G}^{\frac{1}{4} (d_1-d_2)-3} C_2 (\frac{1}{4} (d_1-d_2)-2) (\frac{1}{4} (d_1-d_2)-1) (d_1-d_2) \phi ^{C_2}+\frac{1}{4} T^{C_3} \mathcal {G}^{\frac{1}{4} (d_1+d_2)-3} C_1 (d_1+d_2) \nonumber \\&\quad \times (\frac{1}{4} (d_1+d_2)-2) (\frac{1}{4} (d_1+d_2)-1) \phi ^{C_2}) \dot{\mathcal {G}}^2+\ddot{\phi } C_3 C_4 (\frac{1}{4} T^{C_3} \mathcal {G}^{\frac{1}{4} (d_1-d_2)-1} C_2^2 (d_1-d_2) \phi ^{C_2-1}+\frac{1}{4} T^{C_3} \mathcal {G}^{\frac{1}{4} (d_1+d_2)-1} C_1 C_2 \nonumber \\&\quad \times (d_1+d_2) \phi ^{C_2-1})+\dot{\phi }^2 C_3 C_4 (\frac{1}{4} T^{C_3} \mathcal {G}^{\frac{1}{4} (d_1-d_2)-1} (C_2-1) C_2^2 (d_1-d_2) \phi ^{C_2-2}+\frac{1}{4} T^{C_3} \mathcal {G}^{\frac{1}{4} (d_1+d_2)-1} C_1 (C_2-1) C_2\nonumber \\&\quad \times (d_1+d_2) \phi ^{C_2-2})+\ddot{T} C_3 C_4 (\frac{1}{4} T^{C_3-1} \mathcal {G}^{\frac{1}{4} (d_1-d_2)-1} C_2 C_3 (d_1-d_2) \phi ^{C_2}+\frac{1}{4} T^{C_3-1} \mathcal {G}^{\frac{1}{4} (d_1+d_2)-1} C_1 C_3 (d_1+d_2) \phi ^{C_2})\nonumber \\&\quad +\ddot{\mathcal {G}} C_3 C_4 (\frac{1}{4} T^{C_3} \mathcal {G}^{\frac{1}{4} (d_1-d_2)-2} C_2 (\frac{1}{4} (d_1-d_2)-1) (d_1-d_2) \phi ^{C_2}+\frac{1}{4} T^{C_3} \mathcal {G}^{\frac{1}{4} (d_1+d_2)-2} C_1 (d_1+d_2) (\frac{1}{4} (d_1+d_2)-1) \phi ^{C_2})\nonumber \\&\quad +2 (\dot{T} \dot{\phi } C_3 C_4 (\frac{1}{4} T^{C_3-1} \mathcal {G}^{\frac{1}{4} (d_1-d_2)-1} C_2^2 C_3 (d_1-d_2) \phi ^{C_2-1}+\frac{1}{4} T^{C_3-1} \mathcal {G}^{\frac{1}{4} (d_1+d_2)-1} C_1 C_2 C_3 (d_1+d_2) \phi ^{C_2-1})+\dot{\mathcal {G}} \dot{\phi } C_3 C_4\nonumber \\&\quad \times (\frac{1}{4} T^{C_3} \mathcal {G}^{\frac{1}{4} (d_1-d_2)-2} C_2^2 (\frac{1}{4} (d_1-d_2)-1) (d_1-d_2) \phi ^{C_2-1}+\frac{1}{4} T^{C_3} \mathcal {G}^{\frac{1}{4} (d_1+d_2)-2} C_1 C_2 (d_1+d_2) (\frac{1}{4} (d_1+d_2)-1) \phi ^{C_2-1})\nonumber \\&\quad +\dot{T} \dot{\mathcal {G}}C_3 C_4 (\frac{1}{4} T^{C_3-1} \mathcal {G}^{\frac{1}{4} (d_1-d_2)-2} C_2 C_3 (\frac{1}{4} (d_1-d_2)-1) (d_1-d_2) \phi ^{C_2}+\frac{1}{4} T^{C_3-1} \mathcal {G}^{\frac{1}{4} (d_1+d_2)-2} C_1 C_3 (d_1+d_2)\nonumber \\&\quad \left. \left. \times (\frac{1}{4} (d_1+d_2)-1) \phi ^{C_2}))\right\} -d_7 \phi ^{d_8}\right] \ge 0, \end{aligned}$$
- WEC
  70 $$\begin{aligned} \rho _{eff}&=\rho +\frac{1}{2\chi }\left[ C_3 C_4 (C_1 T^{C_3} \phi ^{C_2} \mathcal {G}^{\frac{1}{4} (d_1+d_2)}+C_2 T^{C_3} \phi ^{C_2} \mathcal {G}^{\frac{1}{4} (d_1-d_2)})+C_1 C_2 C_3 T^{d_3}+d_4 T^{d_5}+d_6 T+2 \rho (C_3 C_4 (C_1 C_3\right. \nonumber \\&\qquad \times T^{C_3-1} \phi ^{C_2} \mathcal {G}^{\frac{1}{4} (d_1+d_2)}+C_2 C_3 T^{C_3-1} \phi ^{C_2} \mathcal {G}^{\frac{1}{4} (d_1-d_2)})+C_1 C_2 C_3 d_3 T^{d_3-1}+d_4 d_5 T^{d_5-1}+d_6)+24H^3 \Big \{C_3 C_4 \dot{T} (\frac{1}{4} C_2 C_3\nonumber \\&\qquad \times (d_1-d_2) T^{C_3-1} \phi ^{C_2} \mathcal {G}^{\frac{1}{4} (d_1-d_2)-1}+\frac{1}{4} C_1 C_3 (d_1+d_2) T^{C_3-1} \phi ^{C_2} \mathcal {G}^{\frac{1}{4} (d_1+d_2)-1})+C_3 C_4 \dot{\phi } (\frac{1}{4} C_2^2 (d_1-d_2) T^{C_3} \phi ^{C_2-1}\nonumber \\&\qquad \times \mathcal {G}^{\frac{1}{4} (d_1-d_2)-1}+\frac{1}{4} C_1 C_2 (d_1+d_2) T^{C_3} \phi ^{C_2-1} \mathcal {G}^{\frac{1}{4} (d_1+d_2)-1})+C_3 C_4 \dot{\mathcal {G}} (\frac{1}{4} C_2 (\frac{1}{4} (d_1-d_2)-1) (d_1-d_2) T^{C_3} \phi ^{C_2} \nonumber \\&\qquad \times \mathcal {G}^{\frac{1}{4} (d_1-d_2)-2}+\frac{1}{4} C_1 (d_1+d_2) (\frac{1}{4} (d_1+d_2)-1) T^{C_3} \phi ^{C_2} \mathcal {G}^{\frac{1}{4} (d_1+d_2)-2})\Big \}+24qH^4\Big \{ C_3 C_4 \frac{1}{4} C_2 (d_1-d_2) T^{C_3} \phi ^{C_2} \nonumber \\&\qquad \left. \times \mathcal {G}^{\frac{1}{4} (d_1-d_2)-1}+\frac{1}{4} C_1 (d_1+d_2) T^{C_3} \phi ^{C_2} \mathcal {G}^{\frac{1}{4} (d_1+d_2)-1}\Big \} \right] \ge 0. \end{aligned}$$

**Figure 4:**
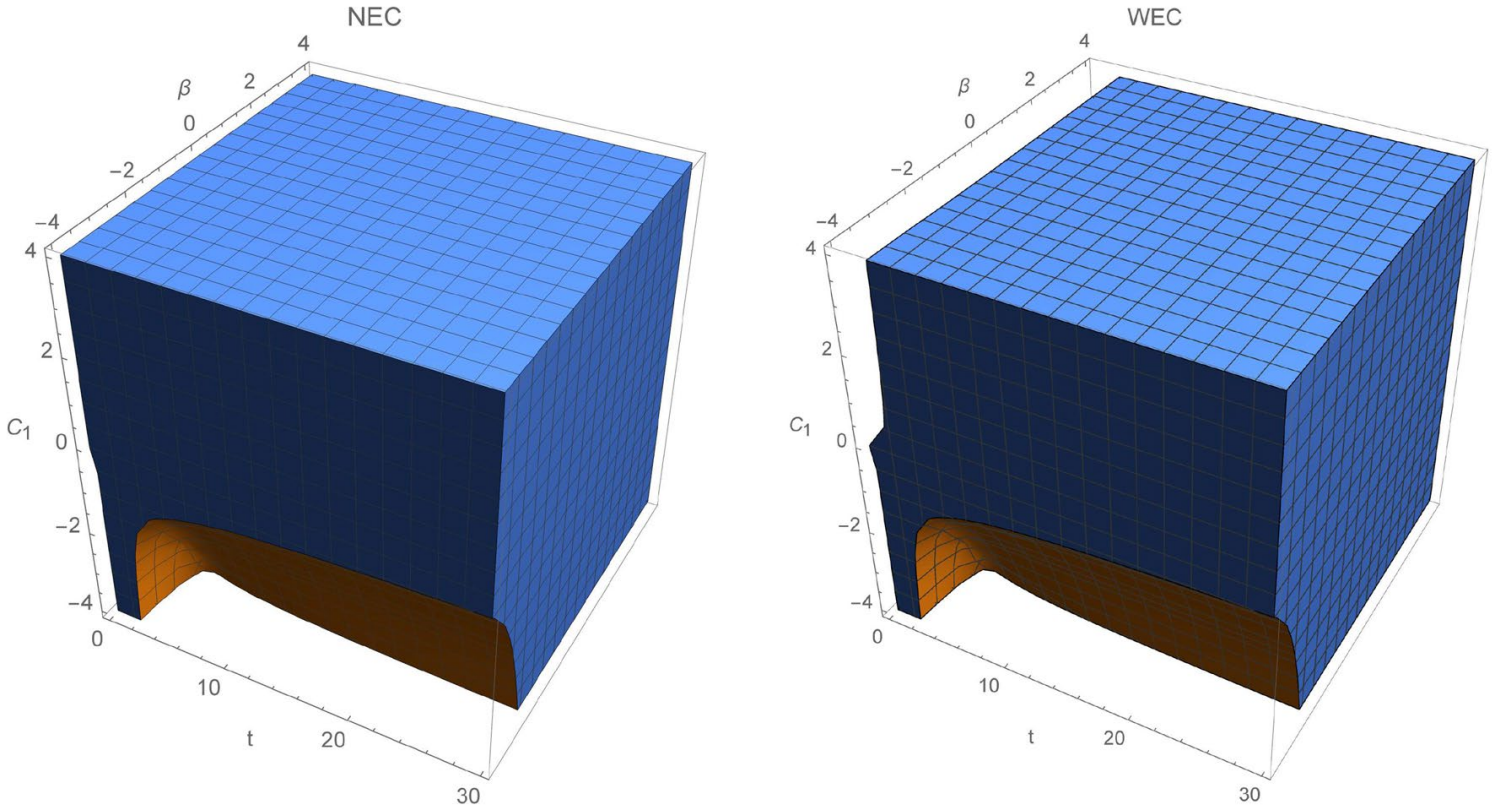
NEC and WEC in power law $f(\mathcal {G},\phi ,T)$ model for $C_2=0.6$ and $C_3=-1$.

**Figure 5:**
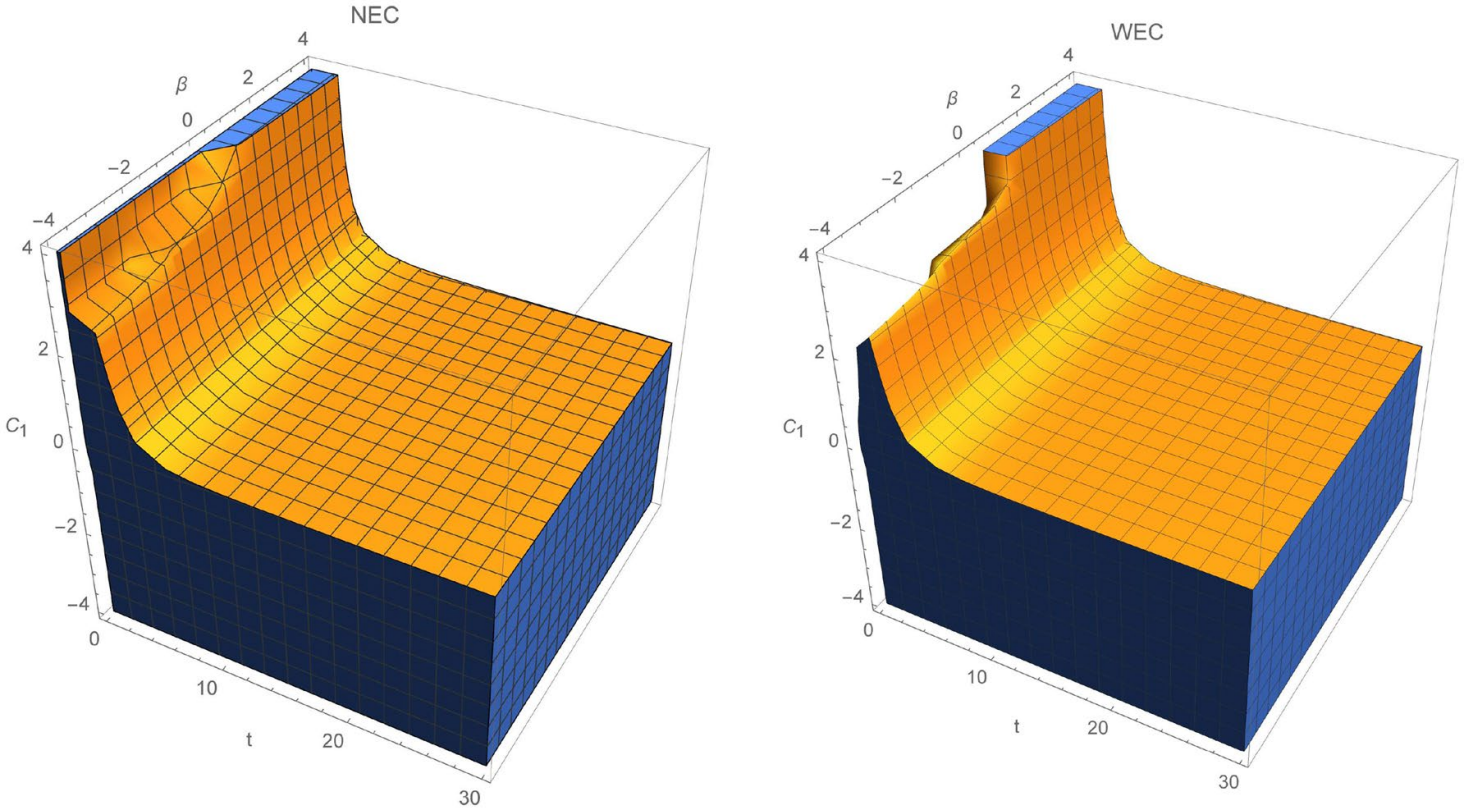
NEC and WEC regions for $C_2=0.3$ and $C_3=0.2$.

**Figure 6:**
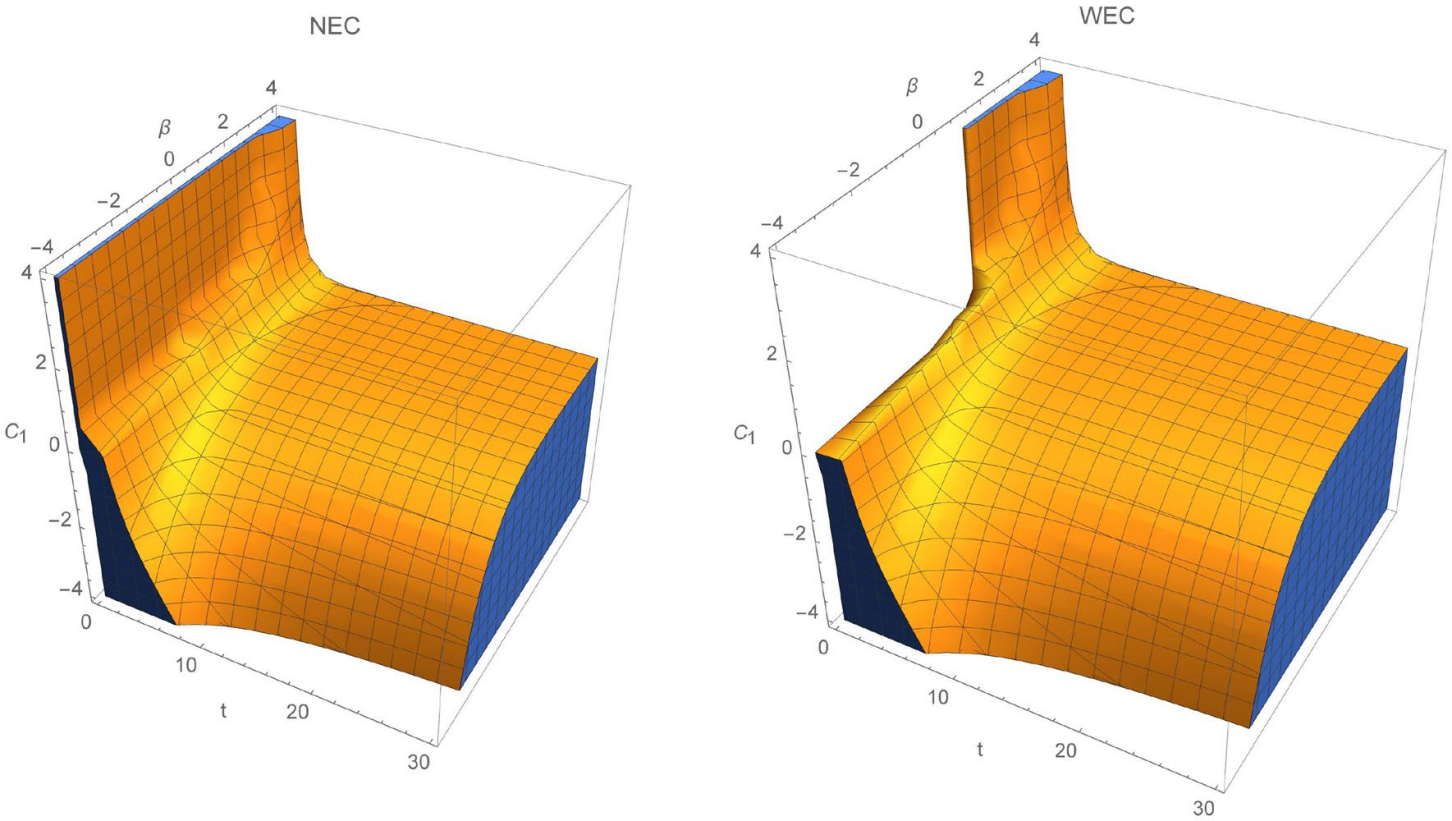
Regions where NEC and WEC are valid for $C_2=-0.5$ and $C_3=-0.25$.

Reconstructed model depends on four integration constants, namely ${C_i}$, *m*, $\beta$ and time. For example, in order to depict graphically the energy contidions we fix constants $C_4=m=1$ and plot regions, where energy conditions hold. Figure 4 describes specific case where $C_2 >0$ and $C_3<0$. In this case, NEC and WEC validity regions coincides, except $C_1>0$ and $t<2$. Another possibility is $C_2>0$ and $C_3>0$, that has been plotted in Fig. 5 for $C_3=0.3$ and $C_3=0.2$. For a given time interval, NEC as well as WEC are shrinking for $C_1>0$ through a given $\beta$ interval. When constant $C_1$ is less than zero, both energy conditions will be satisfied for any time from a given interval. Moreover, there is a region where for $t<2$ only NEC is satisfied when $\beta <0.5$. Next discussed possibility is the case with both $C_2,\; C_3<0$, which is presented in Fig. 6. As time increases both NEC as well as WEC are decreasing. Again, for $C_1>0$ and $\beta <1.2$ at early times, there is a cutoff when NEC holds but WEC not. Interestingly, both NEC and WEC will hold through whole time and $\beta$ parameter intervals for $C_1<0$.

## Specific models

In this section we will discuss energy conditions for the specific case of the $f(R,\phi ,X)$ gravity^36^. We note that this is particular form of the $F(R,\mathcal {G},\phi ,X,T)$ theory, briefly discussed in section I. This is another extension of the scalar–geometry coupling, in which interesting cosmological models have been obtained^36^. We note that the energy conditions for the theories of such type were extensively studied in literature before^59,68^. In manner similar to the Zubair’s work, we also introduce simple $f(\mathcal {G},\phi ,X,T)$ model in the last subsection.

Authors of^36^ considered the Brans–Dicke-type models, where $f(R,\phi ,X)=\gamma (\phi ,X)R$ and corresponding action is:

71 $$\begin{aligned} S=\frac{1}{2\chi }\int d^4x\sqrt{-g}\left[ \gamma (\phi ,X)R\right] + \int d^4x\sqrt{-g} \mathcal {L}_{m}, \end{aligned}$$

with convention $X=-(\nabla \phi )^2/2$. This is one of the most common forms of the scalar–tensor gravity^32^. Field equations for the FLRW metric, after some manipulations are:

72 $$\begin{aligned} 3H^2=\chi \rho _{eff}, \; -(2\dot{H}^2+3H^2)=\chi P_{eff}, \end{aligned}$$

where $\rho _{eff}$ and $P_{eff}$ are given by:

73 $$\begin{aligned}&\rho _{eff}=\frac{1}{f_R}\left[ \rho +\frac{1}{\chi } \left[ 2Xf_X+\frac{1}{2}(f-Rf_R)-3H\partial _tf_R \right] \right] , \end{aligned}$$

74 $$\begin{aligned}&P_{eff}=\frac{1}{f_R}\left[ P-\frac{1}{\chi }\left[ \partial _{tt}f_R+2H\partial _t f_R-\frac{1}{2}(Rf_R-f) \right] \right] . \end{aligned}$$

Function $\gamma (\phi ,X)$ is composed from the kinetic term (*X*) and scalar potential (*V*)^36^:

75 $$\begin{aligned} \gamma (\phi ,X)=X(\phi )-V(\phi ), \end{aligned}$$

and leads to following expressions based on Eqs. (73, 74):

76 $$\begin{aligned}&\rho _{eff}=\frac{1}{\left( X(\phi )-V(\phi )\right) }\left[ \rho +\frac{1}{\chi } [2XR-3H\partial _t\left( X(\phi )-V(\phi )\right) \right] , \end{aligned}$$

77 $$\begin{aligned}&P_{eff}=\frac{1}{\left( X(\phi )-V(\phi )\right) }\left[ P-\frac{1}{\chi }\left[ \partial _{tt}\left( X(\phi )-V(\phi )\right) +2H\partial _t\left( X(\phi )-V(\phi )\right) \right] \right] . \end{aligned}$$

### de-Sitter

For the de-Sitter spacetime considered by Bahamonde et al.^36^, the scalar field was associated with the cosmic time, i.e. $\phi =t$. The corresponding function $\gamma$ is:

78 $$\begin{aligned} \gamma (t)=e^{-H_0t}C_1cos\sqrt{2}H_0(t-t_0), \end{aligned}$$

provided with kinetic term and scalar potential:

79 $$\begin{aligned} X(t)= -\frac{C_1}{2\sqrt{2}}e^{-H_0t}sin\sqrt{2}H_0(t-t_0)-\frac{\chi }{12H_0^2}e^{-3H_0t}, \end{aligned}$$

80 $$\begin{aligned} V(t)=-\frac{C_1}{4}e^{-H_0t}\left[ 4cos\sqrt{2}H_0(t-t_0)+\sqrt{2}sin\sqrt{2}H_0(t-t_0) \right] -\frac{\chi }{12H_0^2}e^{-3H_0t}, \end{aligned}$$

where $C_1$ and $t_0$ are constants of integration. Using above expressions, together with Eqs. (51) and (54), we can obtain expressions for the energy conditions:

- NEC
  81 $$\begin{aligned} \rho _{eff}+P_{eff}=\frac{1}{\chi }\left[ e^{-3 H_0 t} (3 C_1 H_0^2 e^{2 H_0 t} (3 \sqrt{2} \sin (\sqrt{2} H_0 (t-t_0))+2 \cos (\sqrt{2} H_0 (t-t_0))) +\chi (\rho _0(1 +\omega )+2))\right] \ge 0, \end{aligned}$$
- WEC
  82 $$\begin{aligned} \rho _{eff} = \frac{1}{\chi }\left[ e^{-3 H_0 t} (3 C_1 H_0^2 e^{2 H_0 t} (3 \sqrt{2} \sin (\sqrt{2} H_0 (t-t_0))+\cos (\sqrt{2} H_0 (t-t_0))) +(\rho _0+2) \chi ) \right] \ge 0. \end{aligned}$$

**Figure 7:**
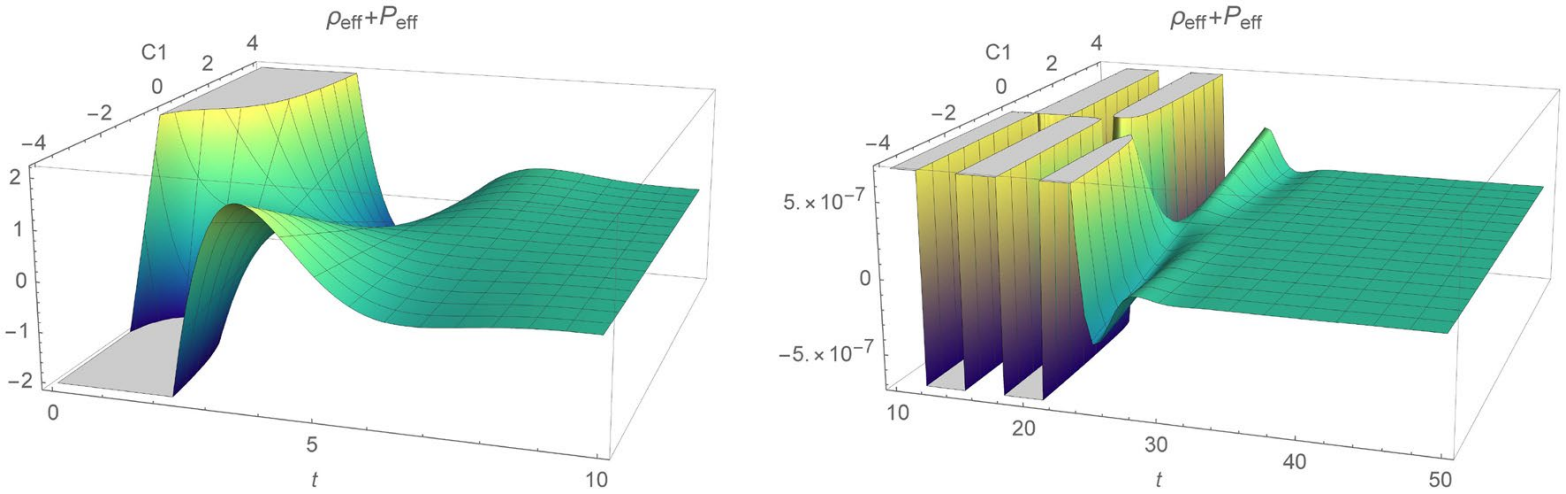
Plot of NEC for $t_0=0$, $\chi =\rho _0=1$ and $C_1\in (-4,4)$ in the de-Sitter reconstructed model.

**Figure 8:**
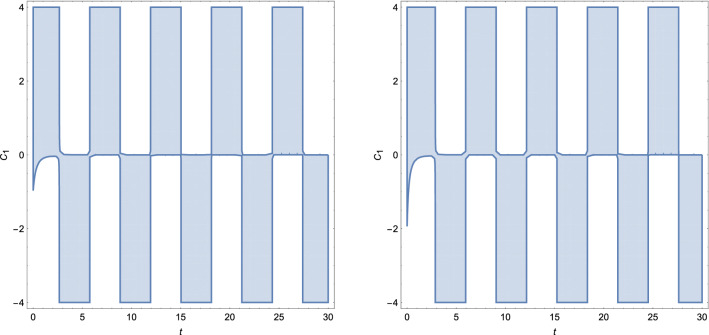
Regions of NEC and WEC validity.

We will discus energy bounds for $n=2/3$ (dust, e.g. $P=0$). For simplicity we take $\chi =\rho _0=1$. The null energy condition has been plotted in Fig. 7 where $t_0=0$. Since, function $\gamma (\phi ,X)$ comes from the damped wave equation, NEC fluctuates and total amplitude diverge to 0 as time parameter increases. When parameter $C_1$ diverts to 0, it promotes increase of the starting values which are either negative ($C_1<0$) or positive ($C_1>0$). Thus, one can conclude that NEC will be satisfied only for the positive parts of oscillations and will converge asymptotically to 0. Regions where the energy conditions are valid are shown in Fig. 8. Changing constant $t_0$ will shift in phase oscillations of $p_{eff}+P_{eff}$ and $\rho _eff$.

### Power law

In this case, also considered by Bahamonde and collaborators in^36^, function $\gamma$ is given by:

83 $$\begin{aligned} \gamma (t)=C_1 t^{p+q}+C_2t^{p-q}, \end{aligned}$$

where:

84 $$\begin{aligned} p=\frac{1-2n}{2},\; q=\frac{\sqrt{1+4n-8n^2}}{2}, \end{aligned}$$

and $\phi =t$. Moreover, kinetic term and potential are:

85 $$\begin{aligned}&X(t)=\frac{1}{4(2n-1)}\left[ C_1(1+2q)t^{p+n}+C_2(1-2q)t^{p-n}-\frac{2}{3N}\chi \rho _0 t^{2-3n}\right] , \end{aligned}$$

86 $$\begin{aligned}&V(t)=\frac{1}{4(2n-1)}\left[ C_1(5-8n+2q)t^{p+n}+C_2(5-8n-2q)t^{p-n}-\frac{2}{3N}\chi \rho _0 t^{2-3n}\right] , \end{aligned}$$

   where $C_i$’s are integration constants. Moreover, null and weak energy conditions are given as:

- NEC
  87 $$\begin{aligned} \rho _{eff}+P_{eff}&=\frac{t^{(-3 n-q-2)}}{N \chi }\left[ (t^q (\rho _0 t^2 \chi (2 n+N \omega +N)-C_1 N (n (5 p+11 q+3) +p^2\right. \nonumber \\&\quad \left. +p (2 q-1)+(q-1) q) t^{3 n+p+q})-C_2 N (n (5 p-11 q+3)+p^2-2 p q-p+q^2+q) t^{3 n+p})\right] \ge 0, \end{aligned}$$
- WEC
  88 $$\begin{aligned} \rho _{eff}=\frac{t^{(-3n-q-2)}}{N \chi }(t^q (\rho _0 t^2 \chi (2n+N)-3 C_1 n N (p+3 q+1) t^{3 n+p+q})-3 C_2 n N (p-3 q+1) t^{3 n+p}) \ge 0. \end{aligned}$$

**Figure 9:**
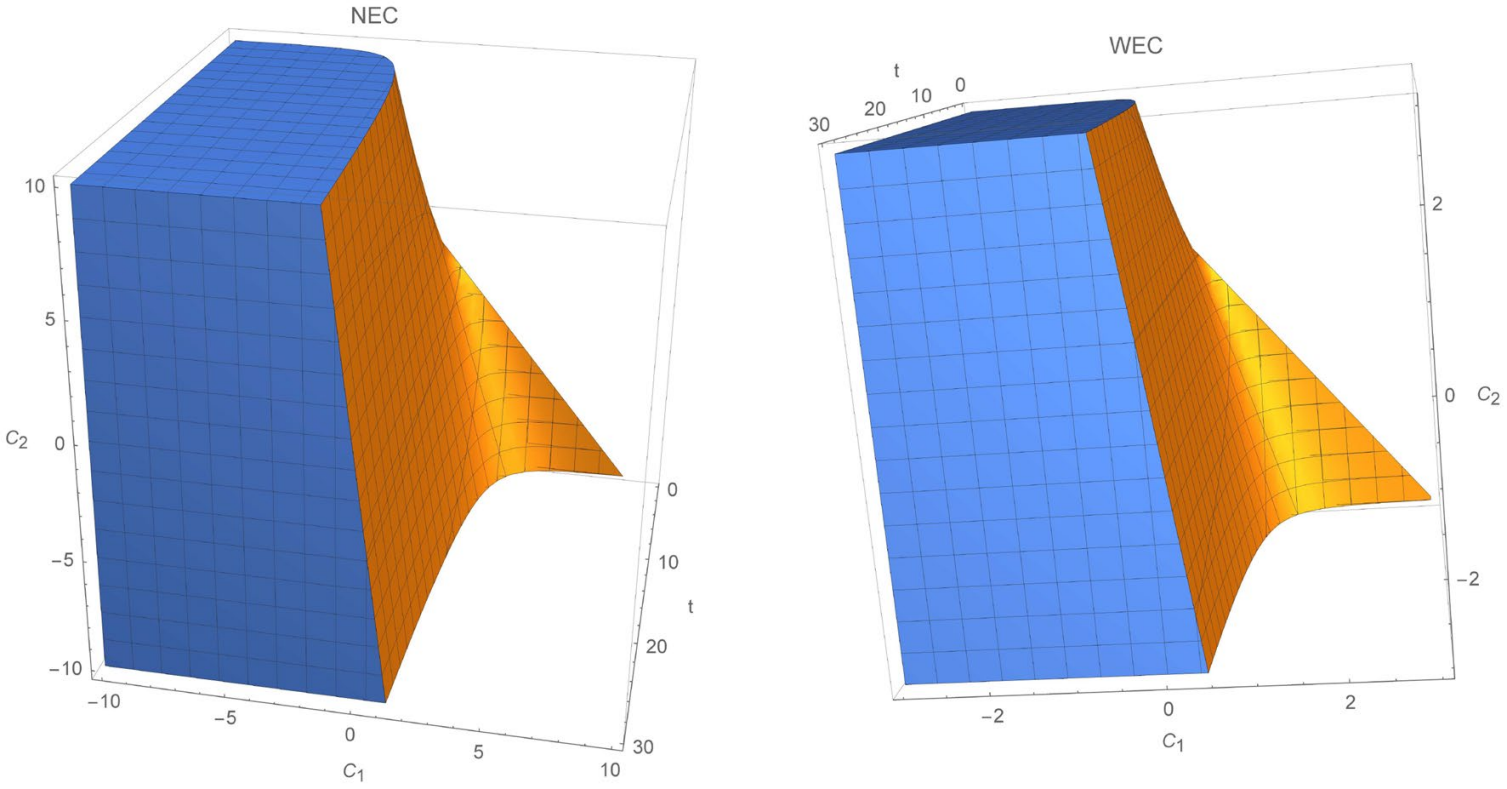
Regions where NEC and WEC are accomplished for power law model.

In this case, the inequalities describing NEC and WEC depend on $\{t,C_1,C_2\}$ (we fix $N=60$), thus energy conditions can be represented in the three dimensional plots. Figure 9 shows regions where NEC and WEC are viable for $t\in (0,30)$, $C_1,\;C_2\in (-10,10)$. In both cases, NEC and WEC regions are getting bigger whenever constant $C_2$ decreases, which can lead to the validity of NEC for later times and positive values of $C_1$.

### $\gamma (\phi ,X)\mathcal {G}$ gravity linearly coupled with matter

Now, we consider the Brans–Dicke type GB gravity linearly coupled to the *g*(*T*) function. This is one of the simplest realizations of the matter–scalar–tensor coupling in the $f\left( \mathcal {G},\phi ,(\nabla \phi )^2,T\right)$ regime. Herein, we consider $g(T)=\mu T^{1/2}$. This is pressureless case of $g(T)=\mu T^{\frac{3w+1}{2(w+1)}}+C_0$ (we fix constant $C_0=0$). Function of this type allows conservation of the energy–momentum tensor, from Eq. (33)^53^. Thus we are working with $f=\gamma (\phi ,X)\mathcal {G}+\mu T^{1/2}$, where again scalar field concides with cosmic time $\phi =t$ and $\gamma (\phi )=X(\phi )-V(\phi )$. From Eqs. (26–28) we obtain field equations for the given model:

89 $$\begin{aligned} X=\frac{-3H^2+\mu T^{1/2}+\chi \rho +12H^3\dot{\gamma }(t)}{\mathcal {G}}, \end{aligned}$$

and

90 $$\begin{aligned} -(2\dot{H}+3H^2)+8H(\dot{H}+H^2)\dot{\gamma }(t)+4H^2\ddot{\gamma }(t)+\frac{\mu }{2} T^{\frac{1}{2}}=0. \end{aligned}$$

Considering power law case, the second equation will be the second order differential equation for unknown function $\gamma (t)$:

91 $$\begin{aligned} \frac{n}{t^2}(2-3n)+8\frac{n^2}{t^2}(n-1)\dot{\gamma }(t)+4\frac{n^2}{t^2}\ddot{\gamma }(t)+\frac{\mu }{2}t^{-\frac{3}{2}n}=0. \end{aligned}$$

Solution of this equation has form:

92 $$\begin{aligned} \gamma (t)=C_1+C_2t^{3-2n}+\frac{(3n-2)}{8n(2n-1)}t^2+\frac{\mu \sqrt{\rho _0}}{2(n+2)(3n-8)n^2}t^{4-\frac{3}{2}n}, \end{aligned}$$

with integration constants $C_1$ and $C_2$. On the other hand, the kinetic term *X*(*t*) is equal to:

93 $$\begin{aligned} X(t)=\frac{C_2 (3-2n)}{2 (n-1)} t^{3-2 n}-\frac{\mu \sqrt{\rho _0} }{8 n^2 (n^2+n-2)}t^{4-\frac{3 n}{2}}-\frac{t^2}{8 n(1-2 n)}+\frac{ \left( \mu \sqrt{\rho _0 t^{-3 n}}+\rho _0 \chi t^{-3 n}\right) }{24 (n-1) n^3}t^4, \end{aligned}$$

and potential is:

94 $$\begin{aligned} V(t)&=\frac{C_2 (3-2n)}{2 (n-1)} t^{3-2 n}-\frac{\mu \sqrt{\rho _0} }{8 n^2 (n^2+n-2)}t^{4-\frac{3 n}{2}}-\frac{t^2}{8 n(1-2 n)}+\frac{ \left( \mu \sqrt{\rho _0 t^{-3 n}}+\rho _0 \chi t^{-3 n}\right) }{24 (n-1) n^3}t^4\nonumber \\&\quad -C_1-C_2t^{3-2n}+\frac{(2-3n)}{8n(2n-1)}t^2-\frac{\mu \sqrt{\rho _0}}{2(n+2)(3n-8)n^2}t^{4-\frac{3}{2}n}. \end{aligned}$$

We note, that procedure described above is another form of the cosmological reconstruction. Once functional form of the theory and the scalar field are specified, one can obtain from the field equations the kinetic term and the scalar potential associated with the discussed cosmological model^36^.

Thus, NEC and WEC are provided:

- NEC
  95 $$\begin{aligned} \rho _{eff}+P_{eff}&= \frac{1}{\chi }\left[ \frac{1}{2 (2 n-1)} (16 C_2 n^2 (4 n^3-13 n+6) t-2 n (12 n^2+n-6) t^{2 n}+5 \mu (2 n-1) \sqrt{\rho _0} t^{\frac{n}{2}+2})t^{-2 (n+1)}\right. \nonumber \\&\quad \left. + 3\frac{n^2}{ t^2}-\frac{\mu }{2}\sqrt{\rho _0 t^{-3n}}\right] \ge 0, \end{aligned}$$
- WEC
  96 $$\begin{aligned} \rho _{eff}=\frac{3}{\chi }\frac{n^2}{t^2}\ge 0. \end{aligned}$$

**Figure 10:**
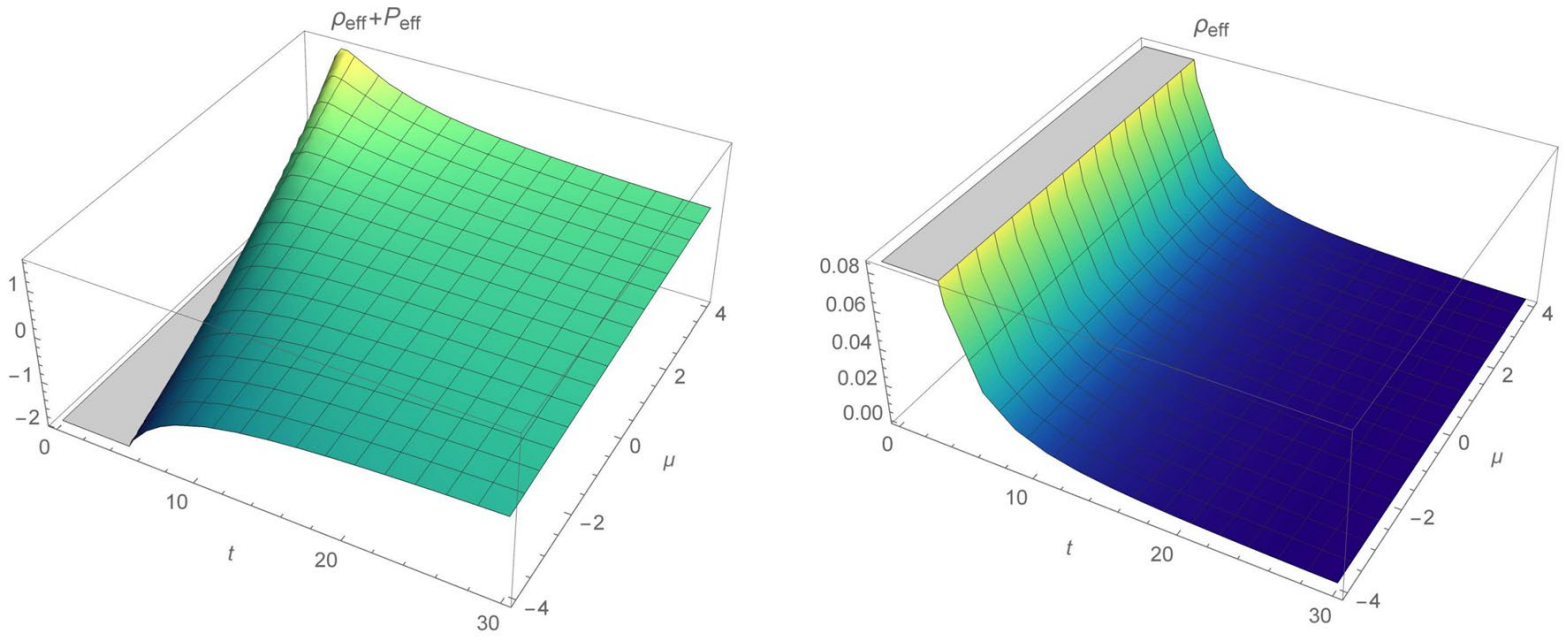
The time evolution of $\rho _{eff}+P_{eff}$ and $\rho _{eff}$ for varying constant $\mu$, where $\rho _{eff}+P_{eff}$ is plotted for $C_2=1$.

**Figure 11:**
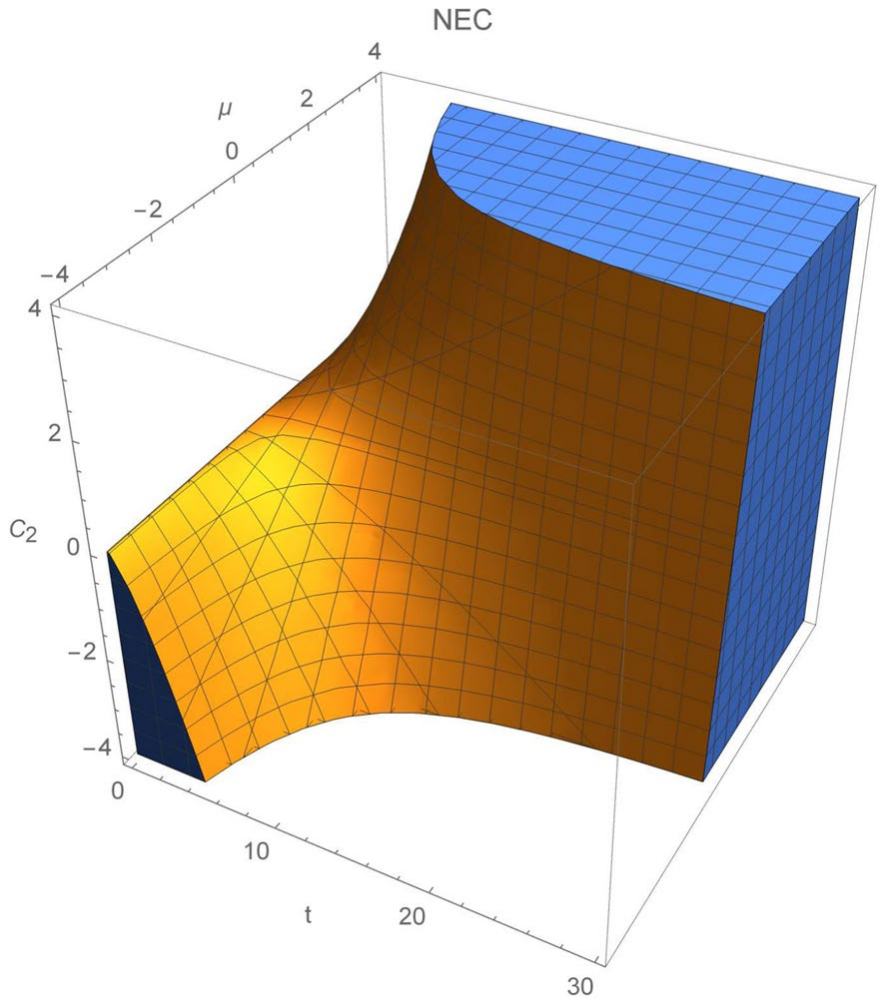
Region where NEC (and WEC) is fulfilled.

**Figure 12:**
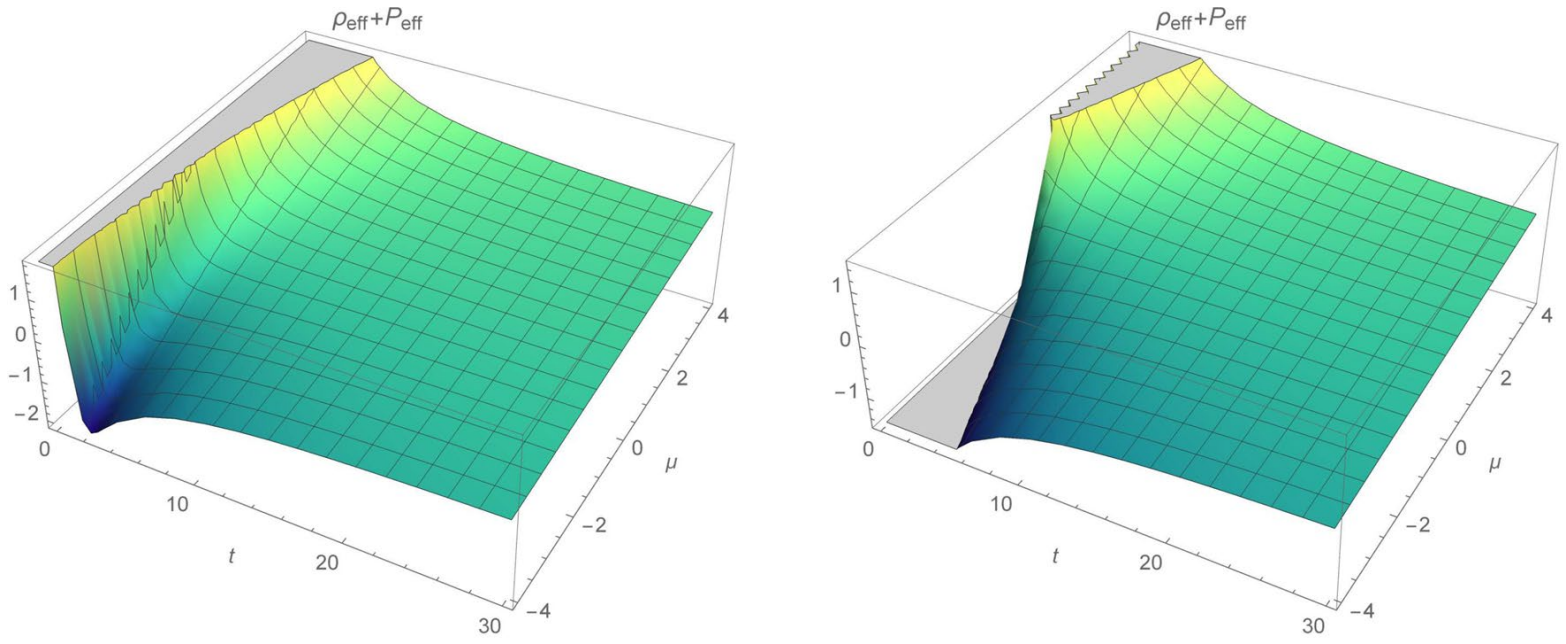
Evolution of $\rho _{eff}+P_{eff}$ as function of time and $\mu$, respectively for $C_2=-0.5$ and $C_2=0.1$.

Clearly, evolution of the effective energy density $\rho _{eff}$ will always be positive for coupling $\chi > 0$. Again we choose for simplicity $\chi =\rho _0=1$ and dust $n=2/3$. We note that the energy conditions will be independent on constant $C_1$ and NEC will depend on $C_2$, $\mu$, *t*. Since, $\rho _eff$ is positive, WEC will hold whenever NEC is fulfilled. Region where energy conditions are positive is increasing with time parameter for $\mu \ge 0$, while decreasing otherwise and is depicted in Figs. 10 and 11. Interestingly, for $C_2<0$ and positive $\mu$, NEC and WEC holds through the whole time parameter interval. We note that for $C_2>0$ and $\mu <0$ energy conditions will always be violated. Figure 12 represents plot of NEC for $C_2<0$ and for $C_2>0$. First case corresponds to $C_2=-0.5$. In this case, evolution of NEC is decreasing with time and for positive $\mu >0$. For the negative values of $\mu$, NEC holds only at early times and reaches its minimum for $t=2.4$. Second figure shows evolution of NEC for $C_2=0.1$. Clearly, as stated before NEC will be violated for negative values of $\mu$ through the given time interval.

## Summary

In the present work we have introduced novel gravity theory, namely the $f(\mathcal {G},\phi ,X,T)$ formalism, which extends the $f(\mathcal {G},T)$ approach toward cases that include arbitrary coupling between following terms: the Gauss–Bonnet term ($\mathcal {G}$), trace of the energy–momentum tensor (*T*), the scalar field ($\phi$) and the kinetic term ($X=(\nabla \phi )^2$). As a results, it is argued that the developed theory may constitute starting point for considerations on the non-minimal matter–scalar field and geometry couplings in the $f(\mathcal {G})$ approaches, which were often studied in the context of modified gravity and low energy actions^41–44,46–49^. We note that, extension introduced here may be interesting in the context of axion fields in the $f(\mathcal {G})$-type theories, in particular for the axion–matter–geometry couplings^50–52^.

In details, the presented theory allowed us to derive field equations from the corresponding action and obtain constrains leading to the conservation of the energy momentum tensor, which is consistent with the $f(\mathcal {G},T)$ theory^68^. Moreover, we have shown that particles follow non-geodesic trajectories, experiencing extra force coming from the non-minimal coupling for the perfect fluid when pressure is non-zero. Using the reconstruction techniques we have also obtained the $f(\mathcal {G},\phi ,T)$ function, that satisfies the de-Sitter and power-law cosmic evolution and discussed energy conditions for this models. Moreover, we have discussed energy conditions for the $f(R,\phi ,X)$ models presented in^36^ for the $\gamma (\phi ,X)R$ functions describing the de-Sitter and power-law scenarios. Assuming specific form of the coupling matter with scalar field and geometry, namely $f=\gamma (\phi ,X)\mathcal {G}+\mu T^{1/2}$, we have obtained $\gamma (\phi ,X)$ which satisfies power-law expansion. We have shown, that for the positive gravitational coupling $\chi$, $\rho _{eff}$ will always stay positive, while for $C_2<0$ and $\mu >0$, NEC and WEC holds at any time.

In conclusions, we have overviewed properties of the reconstructed models of the $f(\mathcal {G},\phi ,X,T)$ and $f(R,\phi ,X)$ gravity showing that NEC and WEC are satisfied in the discussed models, where suitable choice of the free parameters and constants is taken. We note that our reconstructed models are one of the wide class of the gravitational actions coming from Eq. (1) and that our formalism opens up new possibilities in studying modified gravity with emphasis on matter–scalar–geometry couplings in the $f(\mathcal {G})$ gravities. Moreover, general reconstruction procedure presented here can be applied to any cosmological model, once suitable Hubble factor is introduced. Future works should be devoted to the linear stability and cosmological viability of the $f(\mathcal {G},\phi ,X,T)$ gravity and consider other models possible in the $f(\mathcal {G},\phi ,X,T)$ extension.
